# Malondialdehyde-acetaldehyde modified macromolecules and resulting autoantibodies in rheumatoid arthritis pathogenesis: a Systematic Literature Review

**DOI:** 10.3389/fimmu.2025.1648290

**Published:** 2025-11-24

**Authors:** Wenxian Zhou, Nozima Aripova, Hannah J. Johnson, Bryant R. England, Cynthia M. Schmidt, Daniel R. Anderson, Jill A. Poole, Tate M. Johnson, Michael J. Duryee, Geoffrey M. Thiele, Ted R. Mikuls

**Affiliations:** 1 Department of Internal Medicine, Division of Rheumatology, University of Nebraska Medical Center, Omaha, NE, United States; 2 Veterans Affairs (VA) Nebraska-Western Iowa Health Care System, Omaha, NE, United States; 3 McGoogan Health Sciences Library, University of Nebraska Medical Center, Omaha, NE, United States; 4 Department of Internal Medicine, Division of Cardiovascular Medicine, University of Nebraska Medical Center, Omaha, NE, United States; 5 Department of Internal Medicine, Division of Allergy and Immunology, University of Nebraska Medical Center, Omaha, NE, United States

**Keywords:** rheumatoid arthritis (RA), malondialdehyde (MDA), malondialdehyde-acetaldehyde (MAA), post-translational modification, autoantibody

## Abstract

**Objective:**

Substantial progress has been made in understanding the involvement of malondialdehyde-acetaldehyde (MAA) adducts in rheumatoid arthritis (RA) pathogenesis. This systematic review synthesizes current evidence on the role of MAA-modified macromolecules and anti-MAA antibodies in the development, manifestation, and progression of RA.

**Methods:**

MEDLINE, EMBASE, the Cochrane Library, Scopus, and SciFinder were searched through May 6, 2025. Studies were screened based on predefined inclusion/exclusion criteria. Study characteristics were extracted, and quality assessments were performed.

**Results:**

MAA-modified proteins and MAA-specific autoreactive B cells are elevated in synovial and lung tissues of RA patients. Anti-MAA antibodies are enriched in RA-derived synovial fluids compared to serum. Serum levels of anti-MAA IgG and IgA are increased prior to RA onset, and though not RA-specific, were higher in RA patients than those with other conditions. Anti-MAA antibodies do not cross-react with other autoantibodies, such as anti-citrullinated protein autoantibodies, and can be detected in sera from seronegative RA patients. Elevated anti-MAA antibody levels correlate with progression of joint, lung, and cardiovascular complications, as well as biologic treatment responses. Human and animal studies have begun to elucidate mechanisms by which MAA and anti-MAA antibody might contribute to inflammatory and fibrotic changes in RA.

**Conclusions:**

This review provides a comprehensive overview of MAA and its involvement in RA pathogenesis. MAA adducts contribute to loss of immune tolerance and promote both inflammation and fibrosis in RA. Given associations of anti-MAA antibodies with RA disease activity and complications, MAA-related pathways hold promise as both biomarkers and treatment targets in RA.

**Systematic Review Registration:**

https://www.crd.york.ac.uk/PROSPERO/, identifier CRD4202454490.

## 1 Introduction

Rheumatoid arthritis (RA) is a systemic autoimmune disease characterized predominantly by synovial inflammation and bone erosion, leading to progressive joint destruction and physical disability (1). Beyond articular disease, RA is commonly associated with systemic complications, such as cardiovascular and pulmonary disease, that substantially contribute to morbidity and premature mortality (2). A number of post-translational protein modifications (PTMs) have been implicated in RA disease pathogenesis, facilitating the development of disease-related inflammation, tolerance loss, and autoimmunity that are central features of RA (3). In addition to citrullinated proteins, which act as targets of highly specific anti-citrullinated protein antibody (ACPA) (4), other PTMs implicated in RA include homocitrulline and acetyl-lysine, which share marked structural similarities to citrulline. Structurally distinct from these PTMs is the malondialdehyde-acetaldehyde (MAA) adduct, which has emerged as an alternative PTM that may also initiate and propagate the development and progression of RA.

**Figure 1:**
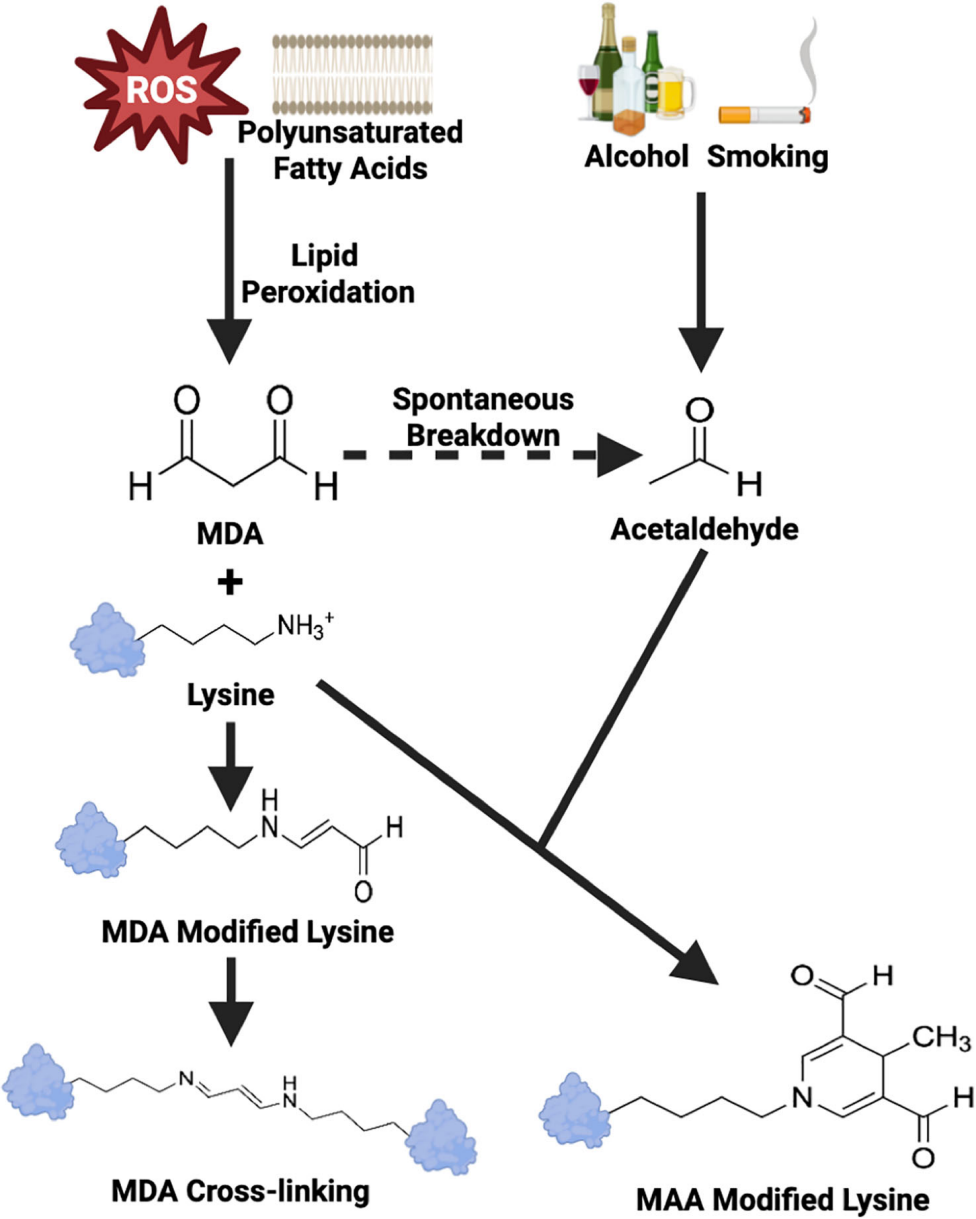
Schematic overview of MDA and MAA adducts formation. ROS, Reactive Oxygen Species; MDA, Malondialdehyde; MAA, Malondialdehyde-Acetaldehyde. Adapted from Grönwall et al. (10).

Systemic inflammation, generated in the context of RA or from other causes such as infection, promotes the formation of reactive oxygen species (ROS) including hydroxyl radical, hydrogen peroxide, and superoxide anions (5). These ROS possess either an unpaired electron or an unstable bond, rendering these molecules highly reactive (6). Polyunsaturated fatty acids, integral components of cell membranes, are particularly susceptible to ROS-mediated damage, a process known as lipid peroxidation (7). Excessive lipid peroxidation compromises cell membrane integrity, disrupts cellular calcium homeostasis, and ultimately leads to cell rupture (7) and the formation of malondialdehyde (MDA) as an byproduct (8, 9). MDA is a highly reactive aldehyde that covalently modifies amino acids with free amine groups. MDA adducted amino acids can further react with one another, forming inter- or intra- molecular cross-links (**Figure 1**) (10). Additionally, MDA reacts with acetaldehyde (from exogenous sources such as alcohol metabolism, cigarette smoke or generated from the spontaneous break down of MDA) to form highly stable MAA adducts (11). MAA is an aromatic ring structure that binds to proteins at the N-terminal amino group or the epsilon amino group of lysine residues, changing a small positively charged amino acid into a bulky neutrally charged MAA adduct. Once formed, MAA adducts have been demonstrated to act as potent immunogens, capable of eliciting strong innate and adaptive immune responses, tolerance loss, and the formation of anti-MAA antibody (12–14). To date, the formation of MAA adducts and anti-MAA immune responses have been implicated in a number of inflammatory and/or fibrotic conditions including cardiovascular disease (15–17), alcohol-induced liver injury (18, 19), and inflammatory bowel disease (20) in addition to RA (21–23). However, no publications to date have systematically reviewed and synthesized existing reports in the literature examining the role of MAA or anti-MAA immunity in the development and/or progression of RA.

Given their potential to both improve prognostication of RA and serve as potential therapeutic targets to improve RA-related outcomes, the objective of this systematic literature review is to provide a comprehensive summary on the reported role of MAA adducts and anti-MAA antibodies in RA. Specifically, we sought to identify studies involving human subjects, animal models, and *in vitro* studies that shed light on how MAA adducts and/or anti-MAA antibodies contribute to the immunogenicity of self-proteins, the generation of disease-related autoimmune responses, and the onset/progression of RA including its extra-articular manifestations.

## 2 Methods

### 2.1 Registration

This systematic review was registered in the PROSPERO database (ID: CRD42024544907) and is reported in accordance with PRISMA 2020 guidelines (24).

### 2.2 Search strategy

To eliminate possible bias in the process of report identification, as other co-authors have participated in relevant research, the search strategy was developed and implemented by an independent research librarian (CMS). MEDLINE (via EBSCOhost), EMBASE (via embase.com, 1974-present version), the Cochrane Library (via Wiley), Scopus, and CAS SciFinder-n were searched through May 6, 2025. Each database search contained two major sections. One section of the search focused on publications containing “MAA” and “rheumatoid arthritis” concepts. The second search section focused on articles containing “MAA”, “arthritis”, and “animal model” concepts. The search concepts were represented by a combination of subject headings (when available) and keywords. Keywords for the “MAA” concept “ were obtained from the Chemical Abstracts Service (CAS) registry records (25), while the keywords for the “RA” concept were derived from the MeSH browser’s entry term list and the EMTREE browser’s synonyms list (26). When available, publication type filters were used to remove conference abstracts and to remove records for review articles and guidelines that were either not published in the Cochrane Library or indexed as systematic reviews or meta-analyses. Because no funds were available for translation, English-language filters were applied. No publication date filters were applied. The search strategy was peer-reviewed by a second member of the University of Nebraska Medical Center medical library’s “systematic review” team. Full search strategies are available in the **Appendix** in the supplementary materials. The initial search was completed on June 5^th^, 2024 and then updated on May 6^th^, 2025. All search results were imported into the project’s EndNote database. EndNote and Zotero duplicate detection tools, as well as manual screens were used to detect duplicate records which were then removed. Citation searching was also conducted during full-text review to identify any additional relevant reports.

### 2.3 Selection criteria

The initial screening process involved eliminating articles based on title and abstract relevance to the research topic, performed by the lead author (WZ). Subsequent full-text assessments for exclusion criteria and relevance were conducted independently by two authors (WZ and MJD). For human studies, only studies that explicitly mentioned both MAA and RA were included. For studies including animal models, the following were considered relevant to RA: collagen induced arthritis, collagen antibody induced arthritis, zymosan-induced arthritis, methylated BSA model, tumor necrosis factor (TNF)-alpha-transgenic mice, K/BxN mice, and SKG mice. *In vitro* studies were included only if antibodies, cells, or tissues examined were derived directly from study participants with RA. Exclusion criteria for full-text articles included clinical case reports/series with fewer than 20 RA patients, studies on herbal or other non-FDA (U.S. Food and Drug Administration) approved medications, narrative reviews, practice guidelines, editorials, and conference abstracts. Any disagreements between reviewers identified during full-text article assessment were discussed and adjudicated by a third reviewer (TRM).

### 2.4 Quality assessment

The quality of evidence from included studies was independently assessed (WZ and MJD) using the Newcastle-Ottawa Quality Assessment Scale (NOS) for cohort studies (27), the NOS adapted for cross-sectional studies, SYRCLE’s Risk of Bias Tool for animal studies (28), and the Quality Assessment Tool for *In Vitro* Studies (QUIN) (29). For clinical studies that included both cross-sectional and cohort components, the cohort component was assessed for quality.

### 2.5 Analysis

For studies involving participants with RA, WZ and NA independently extracted relevant available study characteristics (i.e., study design, country of origin, sample size, age, sex distribution, and comparator population(s)), MAA adduct levels, anti-MAA antibody levels, types of cells/tissues analyzed, and factors significantly associated with anti-MAA antibody levels observed, in addition to summarizing other primary findings from each report. For animal and *in vitro* studies, data on country of origin, animal model type, cell lines, MAA adduct levels, and anti-MAA antibody levels were recorded, in addition to summarizing other primary findings from each report. Given the heterogeneity of reports identified, a meta-analysis was not performed.

## 3 Results

### 3.1 Article selection

**Figure 2:**
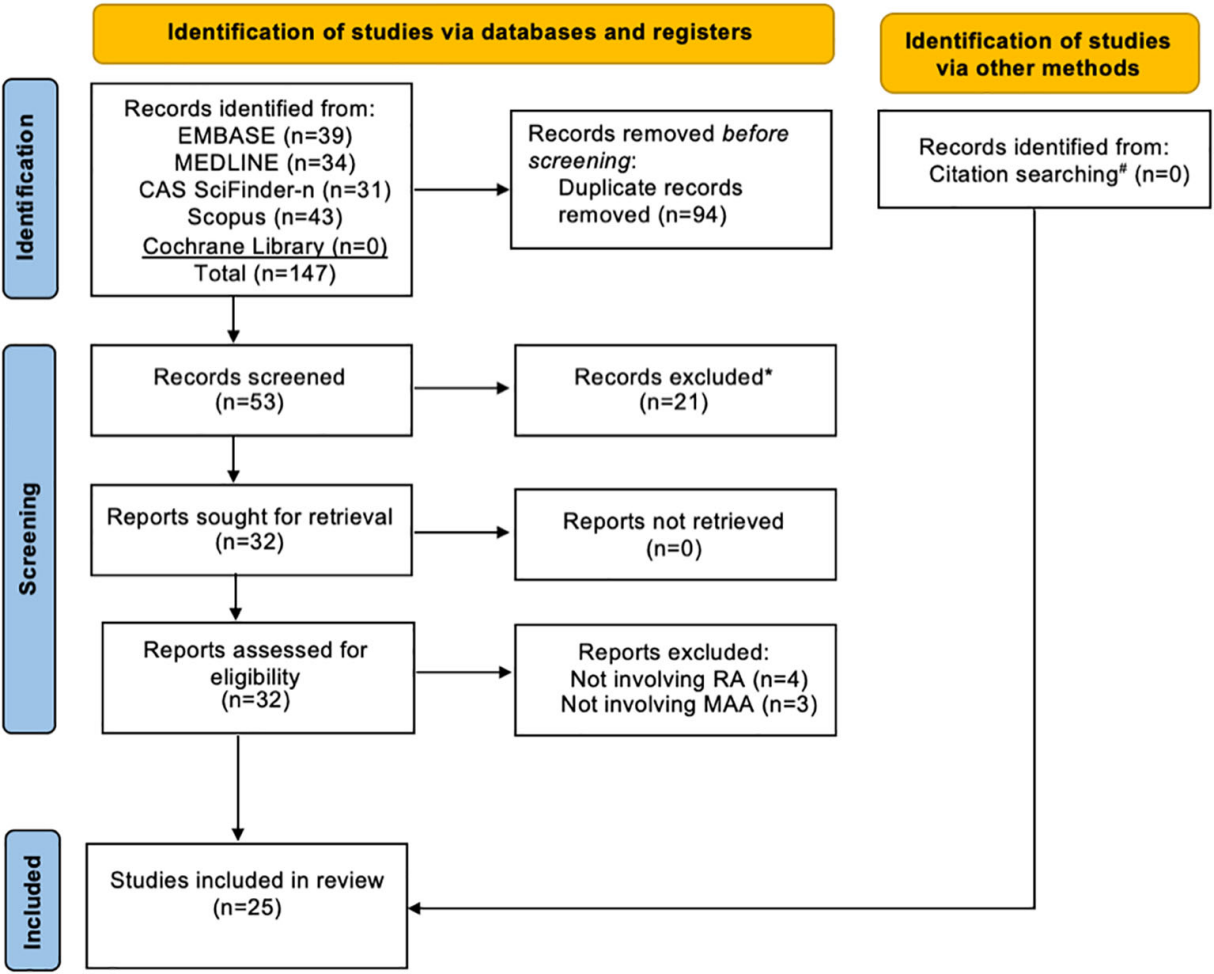
Flow diagram of study selection. RA, Rheumatoid Arthritis; MAA, Malondialdehyde-Acetaldehyde. *All records excluded after the initial title and abstract screening because the research topic did not involve MAA in RA. #Backward citation searching was conducted during full-text review and found no additional relevant reports.

A total of 147 records were identified through initial searches: 39 from EMBASE, 34 from MEDLINE, 31 from CAS SciFinder-n, 43 from Scopus, and none from the Cochrane Library. Of these, 94 were removed as duplicates, and the remaining 53 articles were screened for title and abstract relevance. Based on this screening, 21 articles were eliminated. Consequently, 32 studies underwent full-text assessment, and inclusion/exclusion criteria were applied, leading to the removal of an additional 7 reports (4 reports not involving RA and 3 reports not involving MAA). Further citation searches revealed no additional relevant studies. Ultimately, 25 studies (21–23, 30–51) were included in this review: 17 clinical studies (21–23, 30–41, 46, 51), 7 *in vitro* or animal model studies (42–45, 47, 48, 50), and 1 study with both of these components (49) (**Figure 2**). Seventeen of the 25 reports identified included co-authors of this systematic review.

### 3.2 Study characteristics

Of the 18 studies including a clinical component, 12 were cross-sectional studies (21–23, 30, 33, 35–38, 40, 46, 51), 3 were cohort studies (31, 34, 49), and 3 included both cohort and cross-sectional analyses (32, 39, 41) (**Table 1**). One cross-sectional study also pooled results from two unique study populations with those from other published reports identified herein to generate a weighted overall frequency for anti-MAA antibody positivity (1). These studies were published between 2015 and 2025 and drew participants from 9 countries across 5 continents. Sample sizes ranged from 26 to 2,739 RA participants, with mean age ranging from 36.8 to 67.5 years, and the proportion of female participants ranging from 4.4% to 79%. Ten of the 18 clinical studies included non-RA comparator groups, which varied in composition across reports, and included healthy controls (21, 30, 31, 33, 37, 38), patients with osteoarthritis (OA) (21, 22, 30, 39, 40), or patients with other rheumatologic conditions (30, 35, 39). The 8 remaining studies were primarily restricted to participants with RA. Four of these studies made comparisons within RA subgroups: 3 that compared those with and without interstitial lung disease (ILD) (23, 41, 51), and another comparing those with and without significant coronary artery calcium (CAC) detected by computed tomography (36). In all studies, anti-MAA antibody levels were quantified in serum and/or synovial fluid using ELISA. Most used MAA-modified human or bovine serum albumin (HSA or BSA) as the coating antigen with additional reports using modified antigens that included low-density lipoprotein (LDL) (32, 35), collagen, fibrinogen, and vimentin (41, 51).

**Table 1:** Characteristics and main findings of clinical studies examining MAA expression and anti-MAA immune responses in rheumatoid arthritis.

Reports investigating local anti-MAA antibody production and MAA-specific B Cell clones			
First author/year (country)	Design	Number of participants (N)	Main findings
Mikuls et al./2017 (USA) (22)*	Cross-sectional	RA (29; n=3 for tissue staining) OA (13; n=3 for tissue staining)	•Synovial fluid anti-MAA (IgG, IgM) higher in RA vs. OA •Anti-MAA (IgG, IgM, IgA) higher in paired synovial fluid vs. serum in RA •Marked co-localization of MAA within ectopic lymphoid tissue in RA synovium with citrulline and CD19+/CD27+ B cells (r >0.75)
Sahlstrom et al./2023 (Sweden) (46)	Cross-sectional	ACPA+ RA (14), ACPA- RA (2), ACPA+ early RA (4), ACPA- early RA (4), ACPA+ risk RA (2)	•8 B cell clones identified from RA-derived bone marrow, lung, and joint tissues reactive to malondialdehyde (MDA) or MAA (7 preferentially bound MAA) •Higher proportion of IgG anti-MAA Ab vs. anti-MDA Ab in RA vs. controls
Reports using anti-MAA antibody levels to discriminate RA from non-RA comparators			
First author/year (country)	**Design**	**Number of participants (N)**	**Main findings**
Thiele et al./2015 (USA) (21)*	Cross-sectional	RA (1720; 80 for case control comparison) Healthy controls (80); OA (3) for tissue studies	•MAA adducts increased in RA vs. OA synovium •Anti-MAA Ab (IgA, IgG, IgM) increased in RA (ACPA+ and ACPA-) vs. Control •Correlations of Anti-MAA with RF and ACPA (beta coefficient 0.33-0.61; p<0.001) variable associations of Anti-MAA Ab with RA disease activity •No cross reactivity with ACPA
Mikuls et al./2018 (USA) (30)*	Cross-sectional	RA (284) OA (330) SpA (50) SLE (88) Healthy controls (82)	•Serum anti-MAA Ab with limited RA specificity (IgG 80% positive in RA, 74% positive in OA & SLE, 14% positive in SpA) •IgA anti-MAA Ab higher in RA vs. other groups •Factors positively associated with anti-MAA Ab include race (IgA, IgG) and current smoking (IgA)
Mikuls et al./2020 (USA) (31)*	Cohort	RA (214) Healthy controls (210)	•Serum anti-MAA (IgA/IgG) Ab increased in RA patients prior to RA diagnosis (vs. matched control) but detected later than ACPA/RF; pre-diagnosis differences in anti-MAA most striking in ACPA+ individuals
Grönwall et al./2021 (Sweden) (33)	Cross-sectional	RA (1985, newly diagnosed) Population controls (480) RA-risk (267) SLE (157)	•Serum anti-MAA (IgG) Ab increased in RA vs. control; not RA-specific •Low correlation with other AMPAs but unique positive correlations with RA disease activity •Not increased in at risk RA •No cross reactivity with ACPA or other RA-autoAb
Kononoff et al./2021 (Finland) (35)	Cross-sectional	RA (53, newly diagnosed) SpA (44) UA (38)	•Serum Ab (IgA, IgG, IgM) to MAA-modified LDL increased in newly diagnosed RA vs. other newly diagnosed groups
Rodriguez-Martinez et al./2023 (Spain, Chile) (38)	Cross-sectional	RA (695) Healthy control (392)	•Serum anti-MAA (IgA, IgG, IgM) antibody positivity (≥95^th^ or 98^th^ percentile in controls) observed in 0.6% to 14.8% of RA participants •Meta-analysis estimated anti-MAA IgG positivity among RA to be 38.5% [95% CI: 20.4%-60.3%]
Van den Beukel et al./2023 (Netherlands) (39)	Cross-sectional/cohort	RA (648) PsA (100) OA (95) Gout (93) Other arthritis (250)	•Serum anti-MAA (IgG) Ab positivity more frequent in RA vs. non-RA •No association of anti-MAA status with *HLA-DRB1* shared epitope •Anti-MAA positivity more common in ACPA (-) participants with HLA-DRB1*03 and associated with radiological progression
de Moel et al./2023 (Netherlands, Japan, South Africa, Canada) (37)	Cross-sectional	ACPA+ RA (439) Healthy controls (311)	•Serum anti-MAA (IgG) Ab consistently detected positive in variable proportions of diverse patients •Highest proportion of anti-MAA positive RA observed in South African participants (attenuated after accounting for total IgG)
Reports correlating anti-MAA antibody levels with disease activity and inflammatory mediators			
First author/year (country)	**Design**	**Number of participants (N)**	**Main findings**
Kononoff et al./2020 (Finland) (32)	Cross-sectional/cohort	RA (63; Comparisons at baseline and 1-year)	•Serum antibodies (IgM, IgG) to MAA-modified LDL positively correlated with disease activity in treatment naïve RA (r = 0.30-0.32; p = 0.021-0.030) •Higher baseline disease activity associated with an increase anti-MAA-LDL (IgM) antibody over 1 year (r = 0.36, p = 0.009)
Petro et al./2021 (USA) (34)*	Cohort	RA (1229; Patients initiating biologics)	•Expanded serum auto-Ab profile (anti-MAA Ab [IgG] in addition to ACPA/RF) predictive of biologic treatment response •Anti-MAA Ab less predictive than ACPA
Afonso et al./2024 (Sweden) (49)	Cross-sectional/cohort	Risk-RA (264) Healthy controls (n=437)	•Among at-risk RA group, serum anti-MAA IgG level correlates with circulating inflammatory mediators prior to and at arthritis onset (r = 0.20-0.56; p < 0.05).
Reports correlating anti-MAA antibody levels with Alveolar bone loss and extra-articular complications			
First author/year (country)	**Design**	**Number of participants (N)**	**Main findings**
Lee et al./2024 (USA) (40)*	Cross-sectional	RA (284) OA (330)	•Serum anti-MAA (IgA, IgG, IgM) Ab positive correlated with serum Ab to periodontal bacteria and associated with alveolar bone loss (IgG, IgM) in RA but not OA
Lomzenski et al./2022 (USA) (36)*	Cross-sectional	RA with high CAC score by CT (≥300 Agatston units) (82) vs. RA without high CAC (79)	•Serum anti-MAA IgA Ab (not IgG/IgM) increased in RA-high CAC vs. RA-low CAC •Positive correlations of IgA anti-MAA with insulin resistance (rho = 0.18, p = 0.03), inverse correlations with HDL (rho = -0.20, p =0.01) •Anti-MAA IgA improved 10-yr risk prediction of high CAC.
England et al./2019 (USA) (23)*	Cross-sectional	RA without ILD (1733) vs. RA with ILD (90); tissue staining in RA-ILD (3), other ILD (3), emphysema (3), normal control (3)	•Serum anti-MAA (IgA/IgM) Ab higher RA-ILD vs. RA alone •RA-ILD odds ratio of 2.5 (individual with 3 positive isotypes vs. 0-1 positive isotype) •MAA antigen co-localized with citrulline, CD19+ cells, type II collagen, and vimentin in RA-ILD
Aripova et al/2024 (USA) (41)*	Cross-sectional/cohort	RA without ILD during follow-up (2489) RA with ILD at baseline (114) RA with new ILD during follow-up (136)	•Increased serum Abs to MAA-modified collagen (IgM), fibrinogen (IgA), and vimentin (IgA/IgG) associated with prevalent RA-ILD •Highest quartile of serum anti-MAA (IgA/IgM) Ab to albumin is associated with two-fold risk of incident RA-ILD
Wheeler et al./2025 (USA) (51)*	Cross-sectional	RA without ILD (1880) RA with ILD (121)	•Peripheral biomarkers (including Abs to MAA-modified albumin (IgA, IgG, IgM), collagen (IgA, IgG), fibrinogen (IgG), and vimentin (IgA, IgG)) improve RA-ILD identification beyond clinical risk factors (AUC 0.739 vs. 0.630, p<0.001)

Ab, antibody, ACPA, anti-citrullinated protein antibodies, AMPA, anti-modified protein autoantibodies, AUC, Area Underneath the Curve, CAC, coronary artery calcium, CT, computed tomography, HDL, high density lipoprotein, ILD, interstitial lung disease, LDL, low density lipoprotein, MAA, malondialdehyde-acetaldehyde, OA, osteoarthritis, PsA, psoriatic arthritis, RA, rheumatoid arthritis, RF, rheumatoid factor, SpA, spondyloarthritis; SLE, systemic lupus erythematosus, SpA, Spondyloarthropathy, UA, undifferentiated arthritis. Anti-MAA antibodies measured using MAA-modified albumin (human or bovine serum) unless otherwise noted. *Denotes including of co-authors from systematic review. Reports are organized based on the primary focus of the study.

Characteristics and findings of the 8 *in vitro* or animal model studies identified are summarized in **Table 2**. Two studies (45, 48) conducted *in vitro* experiments investigating how MAA adducts might promote macrophage-fibroblast crosstalk. One study (49) conducted both *in vitro* and animal studies investigating the effect of RA-derived anti-MAA antibody in priming macrophage inflammatory responses, while five studies (42–44, 47, 50) focused on experiments leveraging the collagen induced arthritis (CIA) mouse model with or without combined inhalant exposures of organic dust extract (ODE) or lipopolysaccharide (LPS). Inhalant exposures were added to the CIA model in these studies with the goal of recapitulating inflammatory and pro-fibrotic changes characteristic of RA-ILD (42, 43). There were no studies investigating MAA in other RA animal models, such as collagen antibody induced arthritis, zymosan-induced arthritis, methylated BSA model, tumor necrosis factor (TNF)-alpha-transgenic mice, K/BxN mice, or SKG mice.

**Table 2:** *In vitro* and animal modeling studies examining MAA expression or anti-MAA immune responses in rheumatoid arthritis.

In vitro		
First author/year (country)	Cells atudied	Main findings relevant to MAA or Anti-MAA Ab
Aripova et al./2023 (USA) (45)*	Activated U937 cells; PBMCs from healthy donors; HFLS from RA and OA comparators	Co-culture of macrophage supernatants generated from stimulation with MAA- and citrullinated fibrinogen (FIB-MAA-CIT) with RA derived HFLS led to an aggressive fibroblast phenotype characterized by increased ECM secretion (vs. supernatants generated from FIB-MAA or FIB-CIT); mediated in part by PDGF-BB secretion from macrophages and JNK, Erk1/2, and Akt signaling in HFLS
Afonso et al./2024 (Sweden) (49)	PBMCs from healthy donors, individuals at risk of RA, and RA patients. Bone marrow macrophages from FcγR/FcγRIIb-deficient and control mice.	Anti-MAA antibody sensitizes TLR on macrophages via FcγRs, both accelerating the subsequent LPS-mediated signaling and inducing an inflammatory macrophage phenotype distinct from LPS stimulation alone. Anti-MAA clones from joints and bone marrow had greater effect than both anti-MAA clones from lungs and anti-MDA clones
Aripova et al./2025 (USA) (48)*	Activated U937 cells; PBMCs from healthy donors and patients with RA-ILD; HLF from healthy donor.	Co-culture of macrophage supernatants generated from stimulation with FIB-MAA-CIT with HLF led to an aggressive fibroblast phenotype characterized by increased ECM secretion (vs. supernatants generated from FIB-MAA or FIB-CIT); mediated in part by PDGF-BB and TGF-β secretion from macrophages and JNK, Erk1/2, Akt, and SMAD2/3 signaling in HLF
Animal modeling		
	Animal model	Main findings relevant to MAA or anti-MAA Ab
Poole et al./2019 (USA) (42)*	Mice: CIA +/- inhalant ODE (DBA/1J)	ODE and CIA+ODE led to highest serum anti-MAA Ab; CIA+ODE led to highest serum ACPA, higher arthritis scores, greater bone loss, and highest ECM production in lungs (vs. CIA or ODE alone); findings more striking in male vs. female mice
Mikuls et al./2021 (USA) (43)*	Mice: CIA +/- inhalant LPS (DBA/1J)	CIA+LPS led to highest serum anti-MAA Ab/ACPA (vs. CIA or LPS alone); MAA and CIT increased/co-localized in lung tissues with CIA+LPS; co-exposure led to increased activated macrophages in airway/increased ECM/increased IL-33
Poole et al./2022 (USA) (44)*	Mice: CIA +/- inhalant ODE/wild type vs. HLA-DR4 transgenic (C57BL/6)	CIA+ODE led to increased MAA expression in lung tissues in HLA-DR4+ vs. wild type mice in addition to increased vimentin
Poole et al./2024 (USA) (47)*	Mice: CIA +/- inhalant LPS; castrated and non-castrated male mice (DBA/1J)	Castration (vs intact mice) of male mice led to reductions in pulmonary inflammation/ECM production, and MAA and CIT expression in lung tissues with CIA+LPS; testosterone supplementation reversed some effects of castration
Afonso et al./2024 (Sweden) (49)	Mice: BALB/c, C57BL/6	Anti-MAA antibodies were not arthritogenic in mice, but altered cytokine and growth factor encoding genes in joints
Poole et al./2025 (USA) (50)*	Mice: CIA +/- inhalant LPS (DBA/1J)	CIA+LPS led to increased activated macrophage/monocyte subpopulations with high MHC class II expression in lung tissues. Depleting these subpopulations with IV clodronate liposome administration reduced lung inflammation and fibrosis, and reduced expression of MAA, CIT, and vimentin in lung tissues.

Ab, antibody, CIA, collagen induced arthritis, CIT, citrullinated, ECM, extracellular matrix, FIB, fibrinogen, FcγR, Fc gamma receptor; HFLS, human fibroblast-like synoviocytes, HLF, human lung fibroblast; LPS, lipopolysaccharides, MAA, malondialdehyde-acetaldehyde, MHC, Major Histocompatibility Complex, OA, osteoarthritis, ODE, organic dust extract, PDGF, platelet-derived growth factor, PBMC, peripheral blood monocyte, RA, rheumatoid arthritis; RA-ILD, rheumatoid arthritis associated interstitial lung disease. *Denotes inclusion of co-authors from systematic review.

### 3.3 Quality assessments

Quality assessments for cross-sectional and cohort studies were analyzed using the NOS tool (**Supplementary Tables 1**, **2**). Five studies were deemed to provide moderate quality of evidence, downgraded due to small sample size (<100 participates per group) and failure to control for patient characteristics such as age and sex, whereas the remaining 13 studies were assessed as providing high quality of evidence. For the 6 animal studies identified, quality assessments were conducted using SYRCLE tool, indicating that all studies satisfied approximately half of the criteria, consistent with a moderate risk of bias (**Supplementary Table 3**). None of the animal studies reported concealed allocation, blinded caregiver intervention, or random assessment. Additionally, most of the animal studies did not report random housing or the use of blinded assessments. The 3 *in vitro* studies met 58%-66% of the criteria on the QUIN tool, indicating a moderate risk of bias (**Supplementary Table 4**). None of the *in vitro* studies reported operator and outcome assessor details, randomization, and blinding.

### 3.4 MAA expression in RA-derived tissues

**Figure 3:**
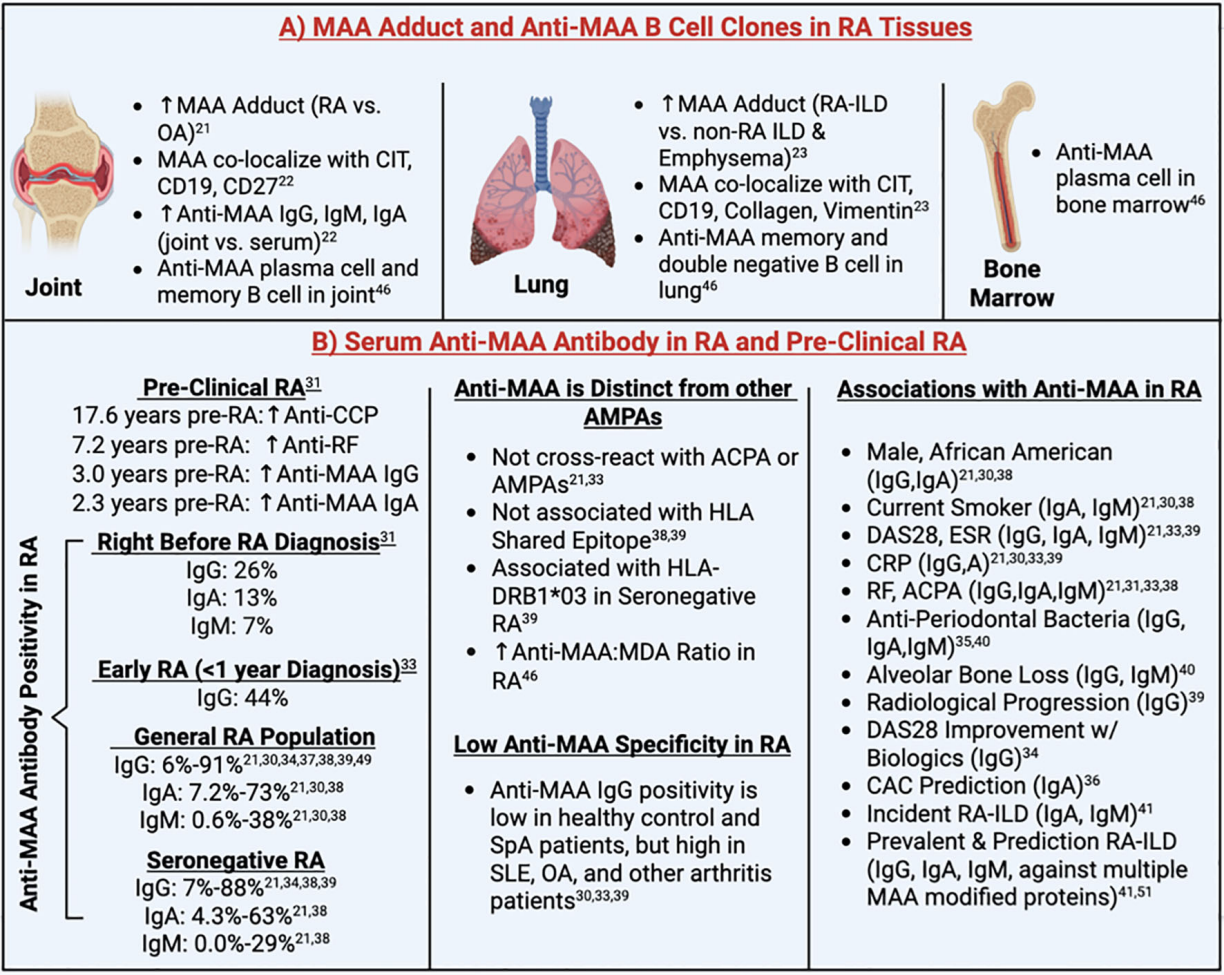
Summary of clinical findings. ACPA, Anti-Citrullinated Protein Antibodies; AMPA, Anti-Modified Protein Autoantibody; CAC, Coronary Artery Calcium; CCP, Cyclic Citrullinated Peptide; CD, Cluster of Differentiation; CIT, Citrulline; CRP, C-Reactive Protein; DAS28, 28-joint Disease Activity Score; ESR, Erythrocyte Sedimentation Rate; HLA, Human Leukocyte Antigen; ILD, Interstitial Lung Disease; MAA, Malondialdehyde-Acetaldehyde; OA, Osteoarthritis; RA, Rheumatoid Arthritis; RF, Rheumatoid Factor; SLE, Systemic Lupus Erythematosus; SpA, Spondyloarthropathy. Superscript are reference numbers.

Results from clinical studies reporting MAA tissue expression and/or anti-MAA antibody concentrations in serum and/or synovial fluid are summarized in **Table 1** and **Figure 3**. Collectively, 3 studies demonstrated increased MAA antigen expression in synovial and lung tissues of RA patients (21–23). Two of these reported increased MAA expression in the synovium of RA patients compared to OA patients (21, 22), while the other study reported increased MAA expression in lung tissues of RA-ILD patients compared to individuals with other forms of ILD or emphysema (23). In addition, MAA was found to co-localize with type II collagen and vimentin in lung tissues of RA-ILD patients, suggesting that these might represent sources of MAA-modified proteins (23). Notably, MAA was also shown to strongly co-localize with citrullinated antigens and CD19+ B cells within ectopic lymphoid tissues in synovial and lung tissues, suggesting a role of MAA in modulating immune responses to citrullinated antigens and promoting local autoantibody production (22, 23).

### 3.5 Local production of anti-MAA antibodies in RA-derived tissues.

Among patients with RA, anti-MAA-albumin IgG, IgA, and IgM antibodies were enriched in the synovial fluid compared to serum, whereas concentrations did not differ in those with OA (22). This suggests the possibility of localized autoantibody production in synovial tissues of RA and is supported by results from the same study demonstrating that MAA-modified proteins co-localize with CD19+ and CD27+ B cells in synovium derived from RA patients (22). Further confirming the local production of anti-MAA antibody, a subsequent study isolated 8 clones consisting of memory B cells, plasma cells, and CD27-IgD-double-negative B cells from synovial fluid, lung and bone marrow of RA patients, which were all highly specific to MAA antigens (46). Notably, two of the three anti-MAA clones isolated from the lung tissues were derived from ACPA+ at risk RA patients (study participants without signs or symptoms of RA), suggesting that the lung may serve as an early site of autoantibody production, before the onset of clinical RA.

### 3.6 Humoral responses to MAA in RA

Anti-MAA antibody status was reported in a subset of clinical studies, with positivity defined using different thresholds (85^th^-99.4^th^ percentile of control populations (21, 30, 31, 33, 37–39) or in the top two tertiles of RA participants (34)) with substantial variability in proportions of seropositive individuals observed. Anti-MAA positivity in RA participants ranged from 6%-91% for IgG (21, 30, 31, 33, 34, 37–39), 7.2%-73% for IgA (21, 30, 38), and 0.6%-38% for IgM (21, 30, 38). A study that pooled data from 9 reports identified in the current review with data from 2 independent study populations estimated the overall frequency of anti-MAA IgG positivity among RA patients to be 38.5% [95% CI: 20.4%-60.3%] (38). Aligning with these results, a subsequent study from the Netherlands reported a prevalence of anti-MAA IgG positivity of 46.1% among 648 participants with RA (39). In addition, anti-MAA IgG was found to be positive in seronegative RA populations (7-88% positivity) (21, 33, 34, 38, 39).

Available data suggest that anti-MAA antibody does not cross-react with other anti-modified protein autoantibodies (AMPAs) and its presence is not associated with *HLA* shared epitope (in contrast to ACPA positivity) (38, 39). Instead, one study found that anti-MAA antibody positivity was significantly associated with HLA-DRB1*03, especially among those with seronegative RA [OR 2.37; 95% CI 1.50, 3.74] (39). Furthermore, a higher ratio of anti-MAA to anti-MDA antibody was observed in the serum of RA patients compared to controls (46), suggesting that MAA is a unique autoantigen and plays a more dominant role than MDA in driving the pathogenesis of RA. Though observed in higher concentrations in RA, even compared to other autoimmune rheumatic conditions, studies have simultaneously shown limited disease specificity of anti-MAA antibodies (30, 33, 39). Serum anti-MAA antibody positivity is low (less than 15%) among healthy controls and spondyloarthrotpathy (SpA) patients, but found in higher levels in other arthritis patients, especially those with systemic lupus erythematosus (SLE) and OA, which limits its utility as a possible diagnostic tool for seronegative RA (30, 33, 39).

### 3.7 Humoral responses to MAA in pre-clinical and early RA

In addition to examining autoantibody concentrations in established RA, reports have examined whether circulating anti-MAA antibodies are increased pre-diagnosis or in individuals deemed to be at high risk of RA development (31, 33, 49). A study using the U.S. Department of Defense Serum Repository reported that circulating anti-MAA antibody (IgA and IgG) levels began to diverge from those of controls approximately 2.3 years and 3 years before the clinical onset of RA, respectively (31). This divergence occurred closer in proximity to disease onset than that of ACPA and rheumatoid factor (RF), suggesting that anti-MAA antibodies may play a role in the later stages of RA initiation. The divergence was also most striking in ACPA positive individuals, suggesting that these autoantibody responses may act together in promoting early disease evolution. Another study from the Epidemiological Investigation of Rheumatoid Arthritis (EIRA) cohort also reported a significant increase in anti-MAA IgG antibody among early RA patients (within 1 year of diagnosis), especially among those who are ACPA positive (33). However, no significant increase in anti-MAA IgG antibody was observed among the RA at risk individuals (ACPA positive individuals without signs of inflammatory arthritis) compared to population-based controls (33). This finding may relate to the previous finding that anti-MAA positivity emerges years after the ACPA positivity is established (31).

Supporting its role in the initial stages of RA development, studies have consistently demonstrated increased anti-MAA antibody concentrations among patients with early RA. In the Karolinska Risk RA prospective cohort, a significantly higher proportion of anti-MAA positivity at RA onset was observed compared to healthy controls (53% vs. 40%, p=0.02) (49). Limited in study power with only 40 RA samples evaluated, a nonsignificant increase (p=0.13) of serum anti-MAA IgG levels were observed at the time of RA onset compared to population controls (49). Indeed, another study using 53 RA samples reported significantly higher anti-MAA-LDL antibodies for IgG, IgM and IgA isotypes among newly diagnosed RA patients compared to other arthritis patients (35). Moreover, serum anti-MAA IgG levels correlate with circulating immune mediators, especially when close to disease onset (49), suggesting that certain IgG anti-MAA clones may contribute to an inflammatory “priming” prior to the onset of clinical symptoms.

### 3.8 Association of anti-MAA antibody levels with clinical factors

Although several reports revealed a positive association of anti-MAA-albumin with other disease characteristics (i.e., inflammatory markers and other autoantibody) and demographics, these associations were not consistent across studies and modest in size when observed (**Table 3**). Three studies investigated the anti-MAA IgG isotype, reporting positive associations with ACPA (33), 28-joint disease activity score (DAS28) (33), erythrocyte sedimentation rate (ESR) (39), and C-reactive protein (CRP) (33, 39), in addition to demonstrating no associations with previous smoking (33, 37). Four separate studies examined all three anti-MAA antibody isotypes, reporting positive associations with RF present for all three isotypes (21, 31) or IgA and IgM only (38). Likewise, ACPA was associated with all three anti-MAA isotypes (21, 31) or IgM only (38) while DAS28 was reported in a single study to be associated with anti-MAA IgA and IgM, but not IgG (21). Similar to associations with autoantibody and composite disease activity measures, studies reported an association of anti-MAA isotype (primarily IgG and IgA) concentrations with other factors such as ESR, CRP levels as well as African American race, male sex, and current smoking status (**Table 3**).

**Table 3:** Reported associations of anti-MAA antibody with disease characteristics of rheumatoid arthritis study participants.

RA outcomes	Association with serum anti-MAA isotypes
Autoantibodies	•RF is associated with either all three isotypes (33), or IgA and IgM only (38). RF is also associated with IgA Anti-MAA-LDL (35) (β=0.21-0.60; p<0.001) •ACPA is associated with either all three isotypes (21, 31), or IgM only (38). One additional report only investigated IgG isotype and found significant association with ACPA (33). ACPA is also associated with IgA Anti-MAA-LDL (35) (β=0.39-0.95; p<0.001)
Acute Phase Reactants	•ESR is associated with either all three isotypes (35), or IgG only (39). ESR is also associated with all three isotypes of Anti-MAA-LDL (35). (β=0.15-0.47; p<0.05) •CRP is associated with either IgG and IgA only (21), or IgA only (30). Two additional report only investigated IgG isotype and found significant association with CRP (33, 39). CRP is also associated with IgG and IgA anti-MAA-LDL (35). (β=0.07-0.35; p<0.05)
Disease Activity	•DAS28 is associated with IgA and IgM only in one study (21). One additional study only investigated IgG isotype and found significant association with DAS28 (33). (β=0.05-0.06; p<0.05)
Demographics	•African American race is associated with IgG and IgA only (30). (β=0.20-0.38; p<0.05) •Male is associated with either IgG only (30, 38), or IgA only (21). (β=0.18-0.27; p<0.05) •Ever smoker is not associated with IgG (33, 37, 38), and associated with IgM only (38). (p<0.01) •Former smoker is not associated with any isotypes (21, 30, 35). •Current smoker is associated with either IgM only (21), or IgA only (30). (β=0.28-0.32; p<0.05)
Alveolar Bone Loss	•Alveolar bone loss is associated with IgG (β=0.53, p=0.012) and IgM (β=0.74, p=0.014) (40).
Radiologic Progression	•Radiographic disease progression is associated with IgG (aOR=1.04, p=0.002) (39).
Coronary Atherosclerosis	•IgA anti-MAA levels improved 10-year prediction of coronary atherosclerosis in RA patients beyond standard cardiovascular risk calculation (C-statistic 0.761 with IgA anti-MAA vs. 0.733 without IgA anti-MAA) (36).
RA-ILD	•Incident RA-ILD is associated with IgA and IgM Anti-MAA-albumin (aHR=1.98-2.13, p<0.05) (51). •Prevalent RA-ILD is associated with anti-MAA-collagen (IgM, aOR=1.28, p=0.03), fibrinogen (IgA, aOR=1.48, p=0.003), and vimentin (IgA/IgG, aOR=1.48-1.78, p <0.003) (41). •Predictive modeling incorporating peripheral biomarkers (including expanded anti-MAA antibody profiling) significantly outperformed clinical risk factor alone in identifying RA-ILD (AUC 0.739 vs. 0.630) (51).

ACPA, anti-citrullinated protein antibody; aHR, adjusted hazard ratio; aOR, adjusted odd ratio; AUC, area underneath the curve; b, b coefficient; CRP, C-reactive protein; DAS28, 28-joint Disease Activity Score; ESR, erythrocyte sedimentation rate; ILD, interstitial lung disease; LDL, low density lipoprotein; MAA, malondialdehyde-acetaldehyde; RA, rheumatoid arthritis; RF, rheumatoid factor. Anti-MAA antibodies measured using MAA modified albumin (human or bovine serum) unless otherwise noted.

### 3.9 Association of anti-MAA antibody levels with alveolar bone loss, treatment response, and RA complications

In addition, two studies reported associations between anti-MAA antibodies and antibodies targeting periodontal bacteria, suggested a link between anti-MAA immune responses and periodontitis complicating the course of RA (35, 40). Lee et al. found that IgG (p=0.012) and IgM (p=0.014) anti-MAA-HSA antibodies were associated with alveolar bone loss in RA patients, associations that were not observed in those with OA (40), suggesting that immune responses to MAA could play a possible pathogenic role in mediating bone loss in the context of RA. Indeed, a cohort study found that higher baseline serum levels of IgG anti-MAA antibodies were associated with greater radiologic disease progression in RA (p=0.002) (39).

In addition to its associations with radiographic progression, anti-MAA antibody assessment may prognosticate other long-term outcomes in RA. A six-month cohort study reported that RA patients positive for ACPA, RF, and anti-MAA IgG antibody were more than twice [95% CI: 1.57-3.51] as likely to achieve DAS28 improvement of >1.2 units and had an average 0.48 [95% CI: 0.26-0.70] units greater DAS28 improvement following treatment with biologic disease-modifying anti-rheumatic drugs (DMARDs), compared to RA patients negative for all three antibodies (34). A separate cross-sectional study demonstrated that IgA anti-MAA antibody improved the 10-year prediction of coronary atherosclerosis in RA patients, above and beyond predictions afforded by a standard cardiovascular risk calculation (C-statistic 0.761 with IgA anti-MAA vs.0.733 without IgA anti-MAA) (36). Moreover, studies conducted in a prospective, multicenter cohort of US Veterans revealed associations between anti-MAA antibody and RA-ILD (23, 41, 51). Serum IgA and IgM antibodies against MAA-modified albumin were higher in RA-ILD vs RA alone and were associated with the subsequent development of incident RA-ILD (23, 41). In addition, increased serum antibodies against MAA-modified collagen (IgM), fibrinogen (IgA), and vimentin (IgA/IgG) were associated with prevalent RA-ILD (41). Individuals positive for all three anti-MAA isotypes have a 2.5-fold [95% CI: 1.29-5.09] higher odds of having RA-ILD than those with 0-1 positive isotypes (23). Moreover, predictive modeling incorporating peripheral biomarkers (including antibodies to MAA-modified albumin (IgA, IgG, IgM), collagen (IgA, IgG), fibrinogen (IgG), and vimentin (IgA, IgG)) significantly outperformed clinical risk factor alone in identifying RA-ILD (AUC 0.739 vs 0.630) (51).

### 3.10 Animal and *in vitro* studies

**Figure 4:**
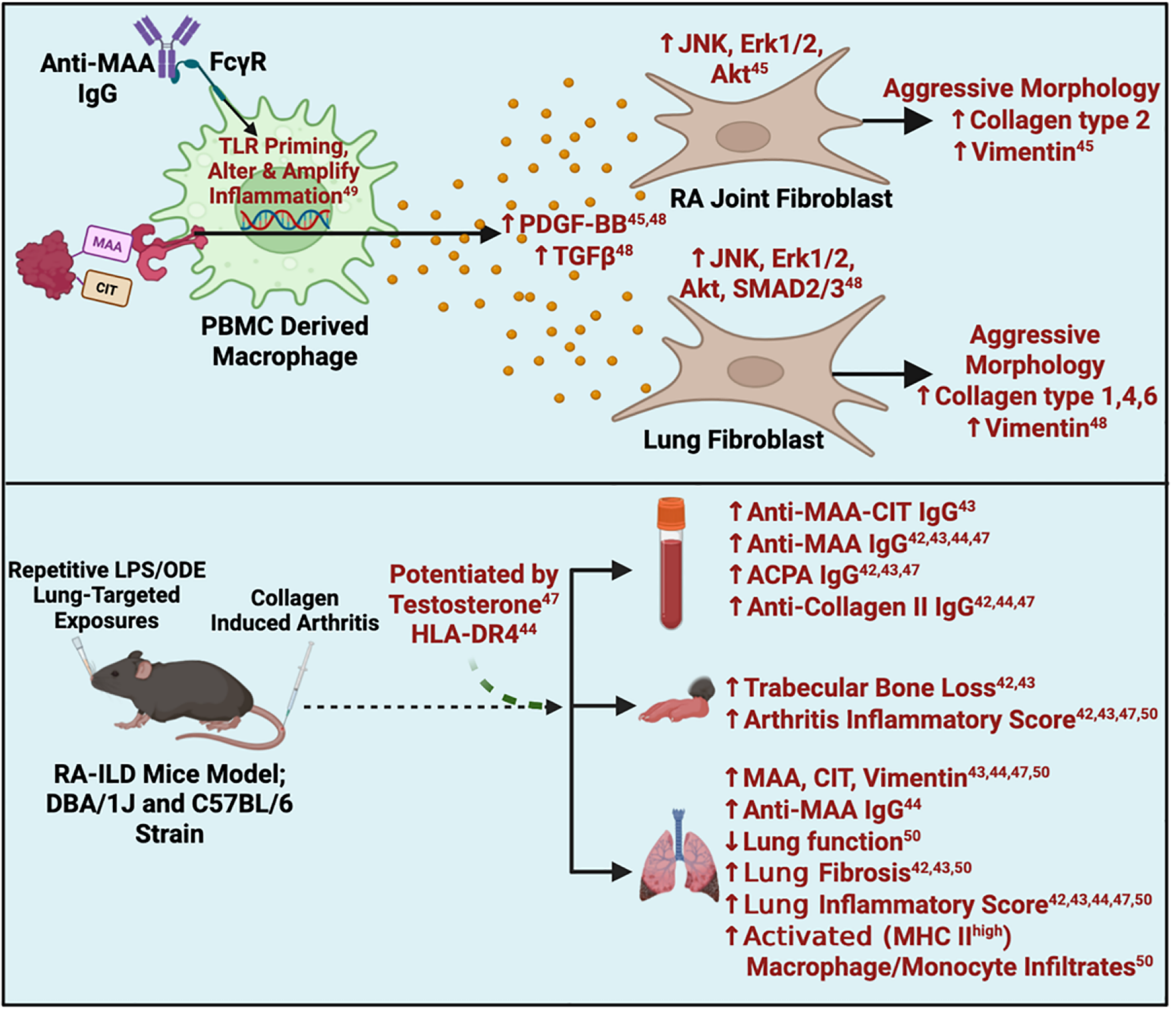
Summary of *in-vitro* and animal findings. ACPA, Anti-Citrullinated Protein Antibodies; Akt, Protein kinase B; CIA, Collagen Induced Arthritis; CIT, Citrulline; ERK, Extracellular Signal-Regulated Kinase; FcyR, Fc gamma Receptor; HLA, Human Leukocyte Antigen; JNK, c-Jun N-terminal kinase; ILD, Interstitial Lung Disease; LPS=Lipopolysaccharide; MAA, Malondialdehyde-Acetaldehyde; MHC, Major Histocompatibility Complex; ODE=Oranic dust extract; PBMC, Peripheral Blood Mononuclear Cell; PDGF, Platelet-Derived Growth Factor; RA, Rheumatoid Arthritis; SMAD, Suppressor of Mother against Decapentaplegic; TGFβ, Transforming Growth Factor Beta; TLR, Toll-like Receptor. Superscript are reference numbers.

Recent efforts have sought to identify mechanistic underpinnings for clinical observations relevant to MAA antigen expression and anti-MAA antibody levels through both animal and *in vitro* studies (**Table 2**, **Figure 4**). Two *in vitro* studies examined the potential role of MAA-modified proteins in promoting an aggressive fibroblast phenotype (QUIN score 58-66%; medium risk of bias) (45, 48). In these studies, investigators noted that fibrinogen co-modified with MAA and citrulline synergistically activates peripheral blood mononuclear cell (PBMC)-derived macrophages, causing significant increases in the release of soluble mediators, such as platelet-derived growth factor BB (PDGF-BB) and transforming growth factor (TGF)-β, compared to stimulation with unmodified fibrinogen or fibrinogen with only a single modification. In turn, these soluble mediators were shown to activate RA human fibroblast-like synoviocytes (HFLS) (via JNK, Erk1/2 and Akt pathways) and human lung fibroblasts (HLFs) via JNK, Erk1/2, Akt, and SMAD2/3 pathways. The activated fibroblasts demonstrated an aggressive phenotype characterized by increased extracellular matrix (ECM) deposition, potentially contributing to the joint destruction and lung fibrosis seen in RA patients (45, 48). In addition to MAA adducts, anti-MAA antibodies were also shown to modulate macrophage activation (QUIN score 58%; medium risk of bias) (49). Anti-MAA IgG antibody sensitizes toll like receptor (TLR) on macrophages via Fc gamma receptors (FcγRs). This sensitization exacerbates subsequent LPS-mediated signaling and induces a unique inflammatory macrophage phenotype, distinct from LPS stimulation alone. In this study, anti-MAA clones isolated from RA joints and bone marrow demonstrated greater inflammatory effects than both anti-MAA clones from lungs and an anti-MDA clone, suggesting clonal diversity. Although systemic administration of anti-MAA antibodies into mice did not lead to arthritis development, unique gene upregulation was observed affecting pathways involved in cytokine activity, lipid binding molecules, and growth factors in the joint tissues of these mice (49). Together, these experiments have shown anti-MAA IgG primes macrophages to have an altered and amplified response toward other inflammatory triggers, which may contribute to the early development of RA (49).

In addition to the studies above, five *in vivo* studies (SYRCLE’s Score 50-60%; medium risk of bias) utilized animal models that combined lung inflammation (induced by inhalation of LPS or ODE) with CIA to recapitulate findings of RA-ILD (**Figure 4**) (42–44, 47, 50). These studies universally demonstrated that mice subjected to LPS/ODE + CIA (vs. LPS/ODE or CIA alone) displayed the highest expression of MAA, citrullinated antigen and vimentin in resected lung tissues, with strong co-localization of MAA with citrulline, similar in magnitude to that reported in human RA-ILD tissues (23) (**Table 2**). Compared to mice receiving one or no treatment, the combined LPS/ODE + CIA exposure resulted in elevated circulating concentrations of ACPA and anti-MAA IgG antibody, as well as higher anti-MAA IgG antibody concentrations in bronchoalveolar lavage fluid (BALF). These LPS/ODE + CIA mice additionally manifested more severe arthritis, trabecular bone loss, increased fibrosis and immune cell infiltrates in lung tissues (42–44, 47). Furthermore, compared to LPS or ODE exposure in isolation, mice exposed to LPS/ODE + CIA demonstrated less neutrophil influx in lung tissues and less inflammatory cytokine/chemokine levels in bronchoalveolar lavage fluid (BALF) (42, 43), suggesting that the addition of arthritis to LPS/ODE inhalant facilitated a transition from a pro-inflammatory to a pro-fibrotic process.

Given the well-established role of *HLA-DR4* in RA pathogenesis (1), one study examined the effects of dual exposure (ODE and CIA) on BALF and lung tissues using *HLA-DR4* transgenic mice (SYRCLE’s Score 60%; medium risk of bias) (44). Compared to wild type C57BL/6 mice with dual exposure, *HLA-DR4* transgenic mice with dual exposure demonstrated increased neutrophils and pro-inflammatory cytokines in BALF. *HLA-DR4* transgenic mice with dual exposure also showed increased MAA and vimentin deposition, CD4+ and CD8+ T cells, and activated macrophages in lung tissues.

Notably, the male-predominance that has been consistently observed in RA-ILD was replicated in CIA mice exposed to inhaled LPS (SYRCLE’s Score 60%; medium risk of bias) (42). Based on these observations and using the same animal model, a separate study investigated the effects of testosterone depletion/repletion in these mice (SYRCLE’s Score 60%; medium risk of bias) (47). In comparison to ‘intact’ mice, castrated LPS-CIA male mice exhibited lower levels of MAA-modified and citrullinated antigens in lung tissues with accompanying reductions in lung inflammation and extracellular matrix production. Testosterone repletion partially reversed several, but not all, of the inflammatory and fibrotic lung endpoints observed in the castrated mice (47).

Lastly, one study examined the role of lung monocytes/macrophages in RA-ILD (SYRCLE’s Score 60%; medium risk of bias) (50). LPS-CIA dual exposure led to increased activated alveolar macrophages, interstitial macrophages, and monocytic-like cells, all with high MHC Class II expression, in lung tissues. These altered macrophage/monocytic cell subpopulations in the LPS-CIA mice were more aligned with CIA than LPS alone. Administration of IV clodronate liposome, known to deplete monocytes, reduced the numbers of alveolar and interstitial macrophages in dually exposed mice, in addition to attenuating lung inflammation and fibrosis, and decreasing levels of MAA, citrulline and vimentin in lung tissues (50).

## 4 Discussion

A growing body of literature now demonstrates that MAA adducts and anti-MAA antibodies appear to play a significant role in the initiation and progression of RA. Illustrating these roles, MAA-modified proteins are enriched in joints of RA patients (vs. OA) (21, 22) and lungs of RA-ILD patients (vs. other forms of ILD or emphysema) (23). Moreover, MAA adducts activate macrophages and fibroblasts *in vitro (*45, 48*)*, potentially driving inflammation and fibrosis in RA-affected tissues. Given that the formation of MAA adducts and anti-MAA immune responses is not RA specific and have been implicated in other conditions such as cardiovascular disease (15–17), alcohol-induced liver injury (18, 19), and inflammatory bowel disease (20), it is quite possible that MAA adducts may also contribute to the development and/or progression of other inflammatory and fibrotic conditions.

In studies of pre-clinical RA, serum levels of anti-MAA IgG and IgA antibodies are increased approximately 3 years before RA disease onset and are higher in patients with established disease compared to healthy controls and patients with alternative rheumatologic diagnoses (30, 31, 33, 35, 39). Beyond serving as potential biomarkers, anti-MAA antibodies appear to play a possible pathogenic role in RA including having the capacity to prime macrophages *in vitro* to amplify inflammatory responses (46). Given that circulating anti-MAA antibodies have been reported in other rheumatologic conditions such as SLE and OA, albeit at lower concentrations than observed in RA, further research is needed to identify whether anti-MAA antibodies might also contribute to disease progression in rheumatic disease s beyond RA.

**Figure 5:**
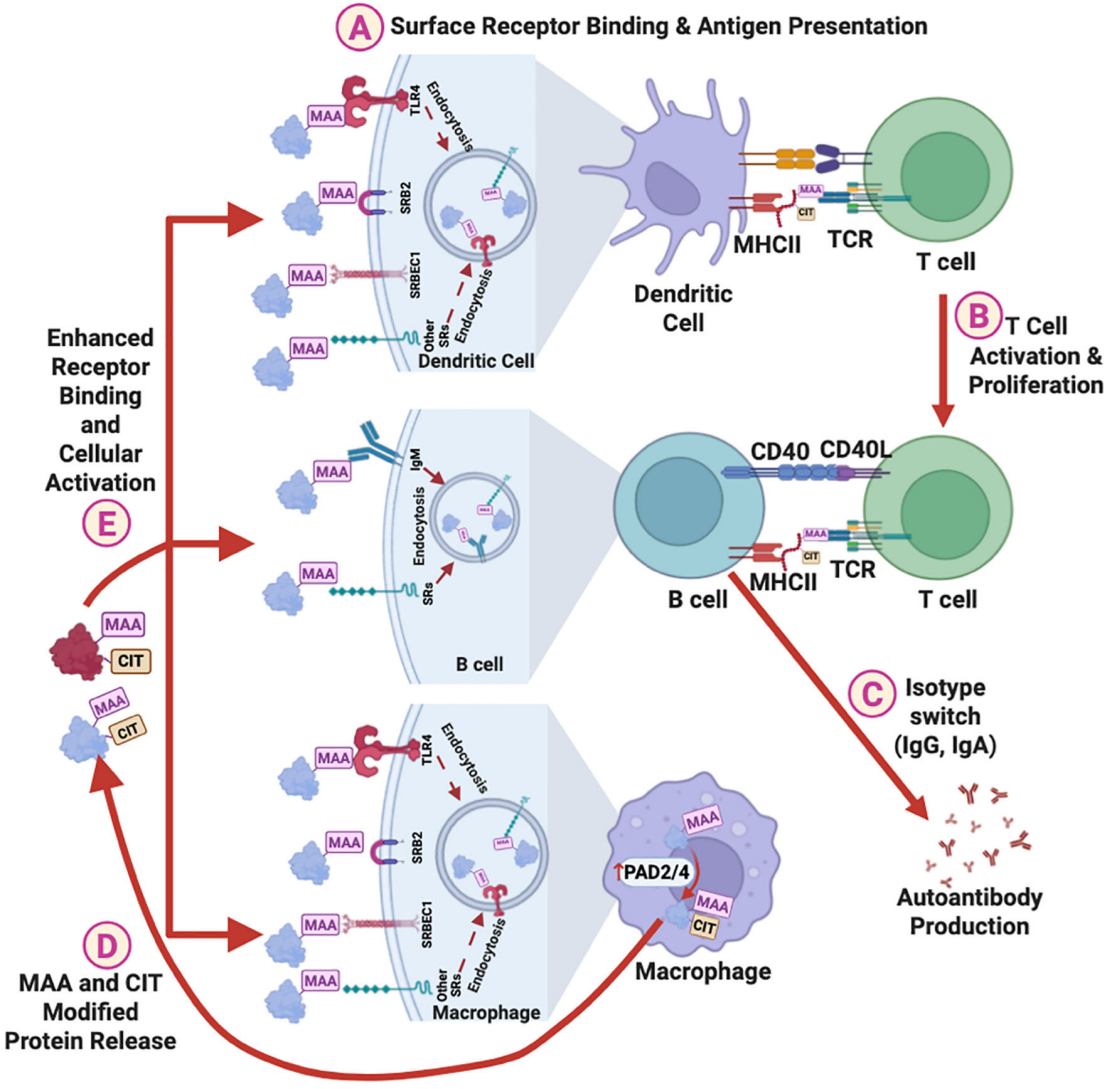
Proposed mechanisms of the role of MAA adduct in promoting autoimmunity. MAA adducts facilitate autoantibody production via following steps: **(A)** Binding to surface receptors on dendritic cells, leading to antigen presentation to helper T cells. **(B)** Activation and proliferation of helper T cells, and **(C)** Activation of B cells and isotype switching, resulting in the production of IgG and IgA anti-MAA antibodies and autoantibodies against MAA-modified self-proteins. In addition, MAA adducts upregulate PAD2/4 expression in macrophages, increasing protein citrullination, and **(D)** promoting the release of MAA and CIT co-modified proteins. **(E)** These MAA and CIT co-modified proteins binds stronger to surface receptors than MAA adducts alone, further enhancing cellular activation and autoantibody production. MAA, Malondialdehyde-Acetaldehyde; CIT, Citrulline; SRs, Surface Receptors; TLR4, Toll-Like Receptor 4; SRB2, Scavenger Receptor Type B2; SRBEC1, Scavenger Receptor Endothelial Cell Type 1; PAD2/4, Protein Arginine Deiminases 2/4; MHC, Major Histocompatibility Complex; TCR, T Cell Receptor.

Emerging evidence suggests that the loss of tolerance in RA may originate at respiratory or oral mucosal sites (52). This hypothesis is supported by the identification of MAA- and citrulline-specific B cell clones in lung tissues of at-risk RA patients (46, 53), as well as studies showing associations between anti-MAA antibody and antibodies targeting periodontal bacteria (35, 40). In addition to studies summarized herein, other recent *in vitro* and animal studies (not involving RA) have provided mechanistic insights into how MAA adducts, a byproduct of inflammation and lipid peroxidation, may facilitate autoantibody generation (**Figure 5**) (54, 55). MAA-adducted proteins bind scavenger receptors and other ligands on antigen-presenting cells (APCs), leading to the presentation of MAA adducts to CD4+ T helper cells (**Figure 5A**) (55). This interaction leads to T cell activation and proliferation (**Figure 5B**), B cell isotype switching, and promotes the production of IgG and IgA autoantibodies (**Figure 5C**). This is supported by a study showing that injecting mice with MAA-modified protein alone leads to the production of anti-MAA IgG autoantibody (17). Moreover, exposure to MAA-modified proteins upregulates PAD2/4 expression in macrophages, leading to enhanced protein citrullination (**Figure 5D**) (54). Proteins that are dually modified with MAA and citrulline bind monocyte scavenger receptors more avidly than antigens that are only MAA-modified or only citrullinated (**Figure 5E**) (55), potentially enhancing the production of both IgG and IgA anti-MAA antibodies and ACPAs.

In addition to not cross-reacting with other autoantibodies, anti-MAA antibody levels have been positively associated with other RA-related autoantibodies (RF, ACPA), disease activity (DAS28), and inflammatory markers (ESR, CRP) (21, 30, 31, 33, 38, 39). Anti-MAA antibody levels also tend to be higher among males, African Americans, and current smokers (21, 30, 33, 37, 38). However, these associations are often modest and sometimes inconsistent—likely due to population heterogeneity, differences in comparator groups, and variations in antibody measurement methods across studies. Further studies evaluating the clinical implications of anti-MAA antibodies in distinct cohorts is warranted.

While anti-MAA antibody levels have been shown to correlate with radiographic disease progression (39), cardiovascular and pulmonary complications (23, 36), and responses to biologic therapies (34), no study to date has investigated their correlation with heart failure in RA. Given that heart failure is the most overrepresented cause of death among RA patients (56, 57), understanding the role of MAA in RA-associated myocardial dysfunction represents a key knowledge gap to be addressed in future work. Additionally, most clinical studies to date have been cross-sectional and focused primarily on the IgG antibody isotype, using MAA-modified albumin as the target antigen. Recent studies reported that associations between anti-MAA antibodies and RA-ILD varies based on both the anti-MAA antibody isotype and protein antigen (41, 51). More prospective cohort studies with larger sample sizes and expanded anti-MAA antibody profiling (e.g., multiple isotypes and MAA-modified tissue-specific proteins and epitopes) are needed to fully assess the diagnostic and prognostic potential of anti-MAA antibodies in RA.

Although MAA adducts and anti-MAA antibodies appear to promote inflammation, bone erosion, and/or fibrosis in RA, no clinical or pre-clinical study to date has evaluated whether pharmacological inhibition of MAA can attenuate RA disease progression. Methotrexate, a first-line RA treatment, has been shown to harbor antioxidant properties and inhibits MAA-adduct formation (58), supporting the potential benefit of directly targeting MAA-related pathways in RA. Recently, reactive aldehyde species inhibitors (RASPi) have been developed to reduce MAA formation in diseased tissues. In addition to recent clinical studies supporting the topical administration of RASPi in the treatment of allergic conjunctivitis (59, 60), other studies have shown that RASPi significantly attenuates alcohol- and smoke- related tissue injuries both in cell culture and in an animal model (18, 61). These hypothesis generating observations underscore the need for future studies to examine whether targeting MAA (with RASPi or through other agents) or MAA-specific downstream pathways could attenuate RA and its complications or prevent the development of RA in high risk individuals.

While this review comprehensively summarized existing literature examining the pathogenic role of MAA adducts and anti-MAA antibodies in RA, there are limitations. This review focused exclusively on studies investigating MAA in RA, excluding those primarily examining MDA. Given that MDA can spontaneously convert into MAA at >10 mM concentration (16), previous MDA-focused studies may have indirectly assessed MAA-related effects. To maintain consistent inclusion criteria, MDA studies were excluded, though such exclusions may have led to the omission of otherwise informative observations (10, 62). In addition, studies included in this systematic review, particularly the *in vivo* and *in vitro* experiments, present moderate risk of bias. Most of these studies did not report sample size calculations, concealed allocation, randomization, blinded interventions, or blinded assessments. Rigorous methodologies in future research are essential to ensure the reproducibility and reliability of findings.

Finally, there was a high degree of heterogeneity among the selected reports, which ranged in design from clinical studies to *in vivo* and *in vitro* investigations. Within the clinical studies, there were cohort studies and cross-sectional studies that included different RA populations with variability in factors including geographic regions represented, RA severity, control groups, and investigated outcomes. Due to the marked heterogeneity of the studies, a meta-analysis was not performed. Illustrative of this heterogeneity, substantial differences have been reported in the prevalence of IgG anti-MAA antibodies in RA ranging from as low as 6% to as high as 91%. These marked differences are likely due to different assay substrates, varying definitions of seropositivity used across studies and a lack of gold- standard assay akin to what is available for the measurement of ACPA or RF where the reproducibility and reliability of the measurements are well defined.

In conclusion, accumulating evidence underscores the potential significance of MAA-modified proteins and anti-MAA antibodies in RA pathogenesis and subsequent disease course. MAA adducts elicit immunogenicity of self-proteins and appear to directly contribute to the development of inflammatory and fibrotic complications of the disease. The association of anti-MAA antibodies with RA disease activity and outcomes underscores their potential as biomarkers for predicting disease progression and complications, as well as potentially guiding therapeutic responses. Therapeutic strategies targeting MAA-adduct formation and biologic pathways engaged following MAA exposure may offer unique benefits in suppressing both inflammation and fibrosis in RA-related complications. However, further investigation is needed to fully harness MAA and anti-MAA antibody as biomarkers for clinical applications and to fully understand the role of MAA and anti-MAA immune responses in the development and/or progression of RA.
