# Supplementary material for: Malondialdehyde-acetaldehyde modified macromolecules and resulting autoantibodies in rheumatoid arthritis pathogenesis: a Systematic Literature Review
Source: Front Immunol. 2025 Nov 24;16:1648290. doi: 10.3389/fimmu.2025.1648290 (PMC12682804; doi:10.3389/fimmu.2025.1648290)
Supplement: Supplementary file 1 [file Table1.docx]

**Supplementary Table 1:** Newcastle-Ottawa Quality Assessment Scale (NOS) for Cross-Sectional Studies.

| **Newcastle- Ottawa Quality Assessment Scale (NOS) for Case Control Studies** | | | | | | | | |
| --- | --- | --- | --- | --- | --- | --- | --- | --- |
|  | **Selection** | | | | **Comparability** | **Outcome** | | **Total** |
| **First Author/ Year** | **Representative of Sample** | **Sample Size** | **Non-Respondents** | **Ascertainment of Exposure** | **Study control for at least two factors** | **Assessment (Secure Record/ Blind Interview)** | **Statistical Test** | **Total Stars (Out of 9)** |
| Thiele et al./ 2015^21^ | ★ | ★ | ★ | ★ | NR | ★★ | ★ | 9 |
| Mikuls et al./ 2017^22^ | ★ | - | ★ | ★ | NR | ★★ | ★ | 6 |
| Mikuls et al./ 2018^30^ | ★ | ★ | ★ | ★ | NR | ★★ | ★ | 7 |
| England et al./ 2019^23^ | ★ | ★ | ★ | ★ | NR | ★★ | ★ | 7 |
| Grönwall et al./ 2021^33^ | ★ | ★ | ★ | ★ | NR | ★★ | ★ | 7 |
| Kononoff et al./ 2021^35^ | ★ | - | ★ | ★ | NR | ★★ | ★ | 6 |
| Lomzenski et al./ 2022^36^ | ★ | - | ★ | ★ | NR | ★★ | ★ | 6 |
| Sahlstrom et al./ 2023^46^ | ★ | - | ★ | ★ | NR | ★★ | ★ | 6 |
| de Moel et al./ 2023^37^ | ★ | ★ | ★ | ★ | NR | ★★ | ★ | 7 |
| Rodriguez-Martinez et al./ 2023^38^ | ★ | ★ | ★ | ★ | NR | ★★ | ★ | 7 |
| Lee et al./ 2024^40^ | ★ | ★ | ★ | ★ | ★ | ★★ | ★ | 8 |
| Wheeler et al./ 2025^51^ | ★ | ★ | ★ | ★ | NR | ★★ | ★ | 7 |

A maximum of two stars was awarded for meeting the comparability criteria: one star for studies that controlled for only one factor, and two stars for studies that controlled for two or more factors. A maximum of two stars was awarded for meeting the outcome assessment criteria: one star for self-report and two star for record linkage or independent blind assessment. A maximum of one star was awarded for meeting each of the other criteria. A higher total number of stars indicates a higher level of evidence and a lower likelihood of bias. A total number of stars greater than 6 indicates a low risk of bias, 4-6 indicates a medium risk of bias, and less than 4 indicates a high risk of bias. NR = Not Reported.

**Supplementary Table 2**. Newcastle- Ottawa Quality Assessment Scale (NOS) for Cohort Studies

| Newcastle- Ottawa Quality Assessment Scale (NOS) for Cohort Studies | | | | | | | | | |
| --- | --- | --- | --- | --- | --- | --- | --- | --- | --- |
|  | **Selection** | | | | **Comparability** | **Exposure** | | | **Total** |
| **First Author/ Year** | **Cohort Representative to Community** | **Non-exposed Cohort is in the same Community** | **Ascertainment (Secure Record/ Structured Interview)** | **Outcome of Interest not present at start of study** | **Study control for at least two factors (Age, Gender)** | **Blind outcome assessment/ Record Linkage** | **Follow-up Long Enough** | **Non-Response Rate, same in two groups** | **Total Stars (Out of 9)** |
| Mikuls et al./ 2020^31^ | ★ | ★ | ★ | ★ | ★★ | ★ | ★ | ★ | 9 |
| Kononoff et al./ 2020^32^ | - | ★ | ★ | ★ | NR | ★ | ★ | ★ | 6 |
| Petro et al./ 2021^34^ | ★ | ★ | ★ | ★ | NR | ★ | ★ | ★ | 7 |
| van den Beukel et al./ 2023^39^ | ★ | ★ | ★ | ★ | NR | ★ | ★ | ★ | 7 |
| Aripova et al./ 2024^41^ | ★ | ★ | ★ | ★ | NR | ★ | ★ | ★ | 7 |
| Afonso et al./ 2024 ^49^ | ★ | ★ | ★ | ★ | NR | ★ | ★ | ★ | 7 |

A maximum of two stars was awarded for meeting the comparability criteria: one star for studies that controlled for only one factor, and two stars for studies that controlled for two or more factors. A maximum of one star was awarded for meeting each of the other criteria. A higher total number of stars indicates a higher level of evidence and a lower likelihood of bias. A total number of stars greater than 6 indicates a low risk of bias, 4-6 indicates a medium risk of bias, and less than 4 indicates a high risk of bias. NR = Not Reported.

**Supplementary Table 3:** Syrcles's Risk of Bias for animal studies.

| **SYRCLE's Risk of Bias for animal studies** | | | | | | | | | | | |
| --- | --- | --- | --- | --- | --- | --- | --- | --- | --- | --- | --- |
|  | **Selection Bias** | | | **Performance Bias** | | **Detection Bias** | | **Attrition Bias** | **Reporting Bias** | **Other** |  |
| **First Author/ Year** | **Random Assignment** | **Similar Baseline** | **Allocation Concealed** | **Random Housing** | **Blind Intervention** | **Random Assessment** | **Blind Assessment** | **All Animal Included** | **Methods & Result Section Match** | **Conflict of Interest** | **Total (Out of 10)** |
| Poole et al./ 2019^42^ | ★ | ★ | No | ★ | No | NR | - | ★ | ★ | ★ | 6 |
| Mikuls et al./ 2021^43^ | ★ | ★ | NR | NR | NR | NR | NR | ★ | ★ | ★ | 5 |
| Poole et al./ 2022^44^ | ★ | ★ | NR | NR | NR | NR | ★ | ★ | ★ | ★ | 6 |
| Poole et al./ 2024^47^ | ★ | ★ | NR | NR | NR | NR | ★ | ★ | ★ | ★ | 6 |
| Afonso et al./ 2024 ^49^ | NR | ★ | NR | NR | NR | NR | NR | ★ | ★ | ★ | 4 |
| Poole et al./ 2025^50^ | ★ | ★ | NR | NR | NR | NR | ★ | ★ | ★ | ★ | 6 |

A maximum of one star was awarded for meeting each of the criteria. The final score was calculated by dividing the total awarded stars by the maximum possible stars. A final score greater than 70% indicates a low risk of bias, 50-70% indicates a medium risk of bias, and less than 50% indicates a high risk of bias. NR = Not Reported.

**Supplementary Table 4:** QUIN Tool for in-vitro Studies.

| **QUIN Tool for in-vitro Studies** | | | | | | | | | | | | | |
| --- | --- | --- | --- | --- | --- | --- | --- | --- | --- | --- | --- | --- | --- |
| **First Author/ Year** | **Clearly States Aims/ Objectives** | **Sample Size Calculation** | **Sampling Technique** | **Comparison Group** | **Method** | **Operator Details** | **Randomization** | **Method of Outcome** | **Outcome Assessor Details** | **Blinding** | **Statistical Analysis** | **Unbiased Results** | **Final Score** |
| Aripova et al./ 2023^41^ | ★★ | NR | ★★ | ★★ | ★★ | NR | NR | ★★ | NR | NR | ★★ | ★★ | 58% |
| Afonso et al./ 2024^49^ | ★★ | NR | ★★ | ★★ | ★★ | NR | NR | ★★ | NR | NR | ★★ | ★★ | 58% |
| Aripova et al./ 2025^48^ | ★★ | ★★ | ★★ | ★★ | ★★ | NR | NR | ★★ | NR | NR | ★★ | ★★ | 66% |

A maximum of two stars was awarded for specifying each criteria (two stars for adequately specified, one star for inadequately specified, and zero stars for not specified). The final score was calculated by dividing the total awarded stars by the maximum possible stars. A final score greater than 70% indicates a low risk of bias, 50-70% indicates a medium risk of bias, and less than 50% indicates a high risk of bias. NR = Not Reported, NA

**Appendix**

**Search strategies:**

**MEDLINE via EBSCOhost**

S4

( S1 OR S2 ) AND LA ("english") NOT ( PT (("review" OR "guideline" OR "practice guideline") NOT ("systematic review" OR "meta-analysis")) ) ) AND PT "Case reports"

Search modes - Boolean/Phrase

S3

( S1 OR S2 ) AND LA ("english") NOT ( PT (("review" OR "guideline" OR "practice guideline") NOT ("systematic review" OR "meta-analysis")) ) ) NOT PT "Case reports"

Search modes - Boolean/Phrase

S2

( ( ( ( TI (AIA OR CAIA OR CIA OR PIA) OR AB (AIA OR CAIA OR CIA OR PIA) OR TI ((experimental OR ACPA OR AIA OR mycobacteri* OR antigen-induced OR anti-citrullin* OR "citrullinated-protein" OR anti-gpi OR antigpi OR "glycoprotein I" OR anti-gp1 OR antigp1 OR "glycoprotein 1" OR "GP I" OR "GP 1" OR GPI OR GP1 OR CII OR Collagen OR Zymosan OR methylated-bsa OR "methylated bovine serum albumin" OR mbsa OR "streptococcal cell wall" OR "bacterial cell wall" OR SCW OR "serum transfer" OR "transferred serum" OR "glucose-6-phosphate isomerase" OR antig6pi OR G6PI OR htnf OR "tumor necrosis factor" OR tnf-tg OR htnf-tg OR tnf* OR Tg3647 OR "k/bxn" OR KRNxNOD OR SKG OR TS1xHACII OR hemagglutinin OR D1CC OR "major histocompatibility complex Class II" OR MHC-II OR MHC-class-II OR "class II MHC" OR TCR OR "t-cell receptor*" OR "organic dust*" OR "agricultural dust*" OR ODE OR pristane) N2(arthriti* OR joint* OR synov* OR cartilag* OR knee* OR "foot pad*" OR foot OR feet OR ankle* OR paw*)) OR AB ((experimental OR ACPA OR AIA OR mycobacteri* OR antigen-induced OR anti-citrullin* OR "citrullinated-protein" OR anti-gpi OR antigpi OR "glycoprotein I" OR anti-gp1 OR antigp1 OR "glycoprotein 1" OR "GP I" OR "GP 1" OR GPI OR GP1 OR CII OR Collagen OR Zymosan OR methylated-bsa OR "methylated bovine serum albumin" OR mbsa OR "streptococcal cell wall" OR "bacterial cell wall" OR SCW OR "serum transfer" OR "transferred serum" OR "glucose-6-phosphate isomerase" OR antig6pi OR G6PI OR htnf OR "tumor necrosis factor" OR tnf-tg OR htnf-tg OR tnf* OR Tg3647 OR "k/bxn" OR KRNxNOD OR SKG OR TS1xHACII OR hemagglutinin OR D1CC OR "major histocompatibility complex Class II" OR MHC-II OR MHC-class-II OR "class II MHC" OR TCR OR "t-cell receptor*" OR "organic dust*" OR "agricultural dust*" OR ODE OR pristane) N2(arthriti* OR joint* OR synov* OR cartilag* OR knee* OR "foot pad*" OR foot OR feet OR ankle* OR paw*)) ) AND (MH "Animals+" OR TI (balb* OR "nu/nu" OR DBA* OR B10* OR C57Bl* OR NOD OR KRNxNOD OR FVB OR "k/bxn" OR SKG OR TS1xHACII OR D1CC-transgenic OR mice OR mouse OR murin* OR murid* OR mus OR rattus OR rat OR rats OR rodent* OR rattus OR wistar OR Lewis OR agouti OR ewe* OR merino OR lamb* OR sheep OR ovine OR ovis OR non-human-primate* OR anthropoid* OR monkey* OR rhesus OR paw*) OR AB (balb* OR "nu/nu" OR DBA* OR B10* OR C57Bl* OR NOD OR KRNxNOD OR FVB OR "k/bxn" OR SKG OR TS1xHACII OR D1CC-transgenic OR mice OR mouse OR murin* OR murid* OR mus OR rattus OR rat OR rats OR rodent* OR rattus OR wistar OR Lewis OR agouti OR ewe* OR merino OR lamb* OR sheep OR ovine OR ovis OR non-human-primate* OR anthropoid* OR monkey* OR rhesus OR paw*) OR MW (balb* OR "nu/nu" OR DBA* OR B10* OR C57Bl* OR NOD OR KRNxNOD OR FVB OR "k/bxn" OR SKG OR TS1xHACII OR D1CC-transgenic OR mice OR mouse OR murin* OR murid* OR mus OR rattus OR rat OR rats OR rodent* OR rattus OR wistar OR Lewis OR agouti OR ewe* OR merino OR lamb* OR sheep OR ovine OR ovis OR non-human-primate* OR anthropoid* OR monkey* OR rhesus OR paw*) ) ) OR TI ("Spontaneous arthriti*" N2 ("non-human primate" OR nhp OR rhesus OR monkey*)) OR AB ("Spontaneous arthriti*" N2 ("non-human primate" OR nhp OR rhesus OR monkey*)) OR (MH "Arthritis, Experimental") OR TI "experimental arthriti*" OR AB "experimental arthriti*" ) ) AND ( ( ( mdhdc OR "71970 43 9" OR "chebi:75949" OR "4-methyl-1,4-dihydro-3,5-pyridinedicarboxaldehyde" OR "3,5-diformyl-4-methyl-1,4-dihydropyridine" OR "4-methyl-1,4-dihydro-3,5-pyridinedicarbaldehyde" OR "4-methyl-1,4-dihydropyridine-3,5-dicarboxaldehyde" OR "mda aa" OR "malondialdehyde acetaldehyde" OR schembl6560442 OR dtxsid10500581 OR q27145654 OR "1,4-dihydro-4-methyl-3,5-pyridinedicarboxaldehyde (aci)" OR "4-methyl-1,4-dihydropyridine-3,5-dialdehyde" OR "4-methyl-1,4-dihydropyridine-3,5-dicarbaldehyde" OR (epitope N2 190359) OR TI ( (maa N4 (adduct* OR anti OR antigen* OR antibod* OR epitope* OR immunoglob* OR igg OR igm OR iga OR ige OR ig OR "immune globulin*" OR modif* OR comodif* OR "post translational" OR alter* OR lysine OR terminal OR adduct* OR albumin OR collagen OR vimentin OR mgp OR "matrix gla protein" OR fibrinogen OR "cross link*" OR autoantibod* OR "self protein*" OR oxidat* OR ldl OR hdl OR vldl OR lipoprotein*)) NOT ((maa OR maas) N1 ("myositis-associated" OR "macro-aggregated albumin" OR "macroaggregated albumin")) ) OR AB ( (maa N4 (adduct* OR anti OR antigen* OR antibod* OR epitope* OR immunoglob* OR igg OR igm OR iga OR ige OR ig OR "immune globulin*" OR modif* OR comodif* OR "post translational" OR alter* OR lysine OR terminal OR adduct* OR albumin OR collagen OR vimentin OR mgp OR "matrix gla protein" OR fibrinogen OR "cross link*" OR autoantibod* OR "self protein*" OR oxidat* OR ldl OR hdl OR vldl OR lipoprotein*)) NOT ((maa OR maas) N1 ("myositis-associated" OR "macro-aggregated albumin" OR "macroaggregated albumin")) ) OR SO ( (maa N4 (adduct* OR anti OR antigen* OR antibod* OR epitope* OR immunoglob* OR igg OR igm OR iga OR ige OR ig OR "immune globulin*" OR modif* OR comodif* OR "post translational" OR alter* OR lysine OR terminal OR adduct* OR albumin OR collagen OR vimentin OR mgp OR "matrix gla protein" OR fibrinogen OR "cross link*" OR autoantibod* OR "self protein*" OR oxidat* OR ldl OR hdl OR vldl OR lipoprotein*)) NOT ((maa OR maas) N1 ("myositis-associated" OR "macro-aggregated albumin" OR "macroaggregated albumin")) ) ) )

Search modes - Boolean/Phrase

S1

( ( mdhdc OR "71970 43 9" OR "chebi:75949" OR "4-methyl-1,4-dihydro-3,5-pyridinedicarboxaldehyde" OR "3,5-diformyl-4-methyl-1,4-dihydropyridine" OR "4-methyl-1,4-dihydro-3,5-pyridinedicarbaldehyde" OR "4-methyl-1,4-dihydropyridine-3,5-dicarboxaldehyde" OR "mda aa" OR "malondialdehyde acetaldehyde" OR schembl6560442 OR dtxsid10500581 OR q27145654 OR "1,4-dihydro-4-methyl-3,5-pyridinedicarboxaldehyde (aci)" OR "4-methyl-1,4-dihydropyridine-3,5-dialdehyde" OR "4-methyl-1,4-dihydropyridine-3,5-dicarbaldehyde" OR (epitope N2 190359) OR TI ( (maa N4 (adduct* OR anti OR antigen* OR antibod* OR epitope* OR immunoglob* OR igg OR igm OR iga OR ige OR ig OR "immune globulin*" OR modif* OR comodif* OR "post translational" OR alter* OR lysine OR terminal OR adduct* OR albumin OR collagen OR vimentin OR mgp OR "matrix gla protein" OR fibrinogen OR "cross link*" OR autoantibod* OR "self protein*" OR oxidat* OR ldl OR hdl OR vldl OR lipoprotein*)) NOT ((maa OR maas) N1 ("myositis-associated" OR "macro-aggregated albumin" OR "macroaggregated albumin")) ) OR AB ( (maa N4 (adduct* OR anti OR antigen* OR antibod* OR epitope* OR immunoglob* OR igg OR igm OR iga OR ige OR ig OR "immune globulin*" OR modif* OR comodif* OR "post translational" OR alter* OR lysine OR terminal OR adduct* OR albumin OR collagen OR vimentin OR mgp OR "matrix gla protein" OR fibrinogen OR "cross link*" OR autoantibod* OR "self protein*" OR oxidat* OR ldl OR hdl OR vldl OR lipoprotein*)) NOT ((maa OR maas) N1 ("myositis-associated" OR "macro-aggregated albumin" OR "macroaggregated albumin")) ) OR SO ( (maa N4 (adduct* OR anti OR antigen* OR antibod* OR epitope* OR immunoglob* OR igg OR igm OR iga OR ige OR ig OR "immune globulin*" OR modif* OR comodif* OR "post translational" OR alter* OR lysine OR terminal OR adduct* OR albumin OR collagen OR vimentin OR mgp OR "matrix gla protein" OR fibrinogen OR "cross link*" OR autoantibod* OR "self protein*" OR oxidat* OR ldl OR hdl OR vldl OR lipoprotein*)) NOT ((maa OR maas) N1 ("myositis-associated" OR "macro-aggregated albumin" OR "macroaggregated albumin")) ) ) AND ( (MH "Arthritis, Rheumatoid+") OR (MH "Arthritis") OR TI (felty* OR "stills disease" OR "still's disease" OR "still disease" OR beauvais* OR caplan* OR rheumarthrit* OR reumarthrit* OR revmarthrit* OR rheumatism* OR reumatism* OR revmatism* OR rheumatoid* OR reumatoid OR revmatoid OR "chronic polyarthriti*" OR "inflammatory arthrit*" OR beauvais OR ((rheumatic OR reumatic OR revmatic OR deform*) N2 (polyarthr* OR arthr*)) OR ( ra NOT ( ((rosmarinic OR "relative abundance" OR retinoic) N2 ra) OR 226ra OR "226 ra" OR radium ) ) ) OR AB (felty* OR "stills disease" OR "still's disease" OR "still disease" OR beauvais* OR caplan* OR rheumarthrit* OR reumarthrit* OR revmarthrit* OR rheumatism* OR reumatism* OR revmatism* OR rheumatoid* OR reumatoid OR revmatoid OR "chronic polyarthriti*" OR "inflammatory arthrit*" OR beauvais OR ((rheumatic OR reumatic OR revmatic OR deform*) N2 (polyarthr* OR arthr*)) OR ( ra NOT ( ((rosmarinic OR "relative abundance" OR retinoic) N2 ra) OR 226ra OR "226 ra" OR radium ) ) ) OR SO (felty* OR "stills disease" OR "still's disease" OR "still disease" OR beauvais* OR caplan* OR rheumarthrit* OR reumarthrit* OR revmarthrit* OR rheumatism* OR reumatism* OR revmatism* OR rheumatoid* OR reumatoid OR revmatoid OR "chronic polyarthriti*" OR "inflammatory arthrit*" OR beauvais OR ((rheumatic OR reumatic OR revmatic OR deform*) N2 (polyarthr* OR arthr*)) OR ( ra NOT ( ((rosmarinic OR "relative abundance" OR retinoic) N2 ra) OR 226ra OR "226 ra" OR radium ) ) ) )

Search modes - Boolean/Phrase

 **************************************************************************************************************

**EMBASE via embase.com, 1974- present version**

No.

Query

Date

#5

(#1 OR #2) AND [english]/lim NOT 'conference abstract'/it NOT (('conference review'/it OR 'review'/it OR 'practice guideline'/de) NOT ([cochrane review]/lim OR [systematic review]/lim OR [meta analysis]/lim)) NOT 'case report'/exp

#4

(#1 OR #2) AND [english]/lim NOT 'conference abstract'/it NOT (('conference review'/it OR 'review'/it OR 'practice guideline'/de) NOT ([cochrane review]/lim OR [systematic review]/lim OR [meta analysis]/lim)) AND 'case report'/exp

#3

#1 OR #2

#2

(mdhdc OR '71970 43 9' OR 'chebi:75949' OR '4-methyl-1,4-dihydro-3,5-pyridinedicarboxaldehyde' OR '3,5-diformyl-4-methyl-1,4-dihydropyridine' OR '4-methyl-1,4-dihydro-3,5-pyridinedicarbaldehyde' OR '4-methyl-1,4-dihydropyridine-3,5-dicarboxaldehyde' OR 'mda aa' OR 'malondialdehyde acetaldehyde' OR schembl6560442 OR dtxsid10500581 OR q27145654 OR '1,4-dihydro-4-methyl-3,5-pyridinedicarboxaldehyde (aci)' OR '4-methyl-1,4-dihydropyridine-3,5-dialdehyde' OR '4-methyl-1,4-dihydropyridine-3,5-dicarbaldehyde' OR (epitope NEAR/3 190359) OR (((maa NEAR/5 (adduct* OR anti OR antigen* OR antibod* OR epitope* OR immunoglob* OR igg OR igm OR iga OR ige OR ig OR 'immune globulin*' OR modif* OR comodif* OR 'post translational' OR alter* OR lysine OR terminal OR adduct* OR albumin OR collagen OR vimentin OR mgp OR 'matrix gla protein' OR fibrinogen OR 'cross link*' OR autoantibod* OR 'self protein*' OR oxidat* OR ldl OR hdl OR vldl OR lipoprotein*)):ab,kw,ti) NOT ((maa OR maas) NEAR/2 ('myositis-associated' OR 'macro-aggregated albumin' OR 'macroaggregated albumin')):ab,kw,ti)) AND ('arthritis'/de OR 'rheumatoid arthritis'/exp OR felty*:ab,kw,ti OR 'stills disease':ab,kw,ti OR 'still?s disease':ab,kw,ti OR 'still disease':ab,kw,ti OR beauvais*:ab,kw,ti OR caplan*:ab,kw,ti OR rheumarthrit*:ab,kw,ti OR reumarthrit*:ab,kw,ti OR revmarthrit*:ab,kw,ti OR rheumatism*:ab,kw,ti OR reumatism*:ab,kw,ti OR revmatism*:ab,kw,ti OR rheumatoid*:ab,kw,ti OR reumatoid:ab,kw,ti OR revmatoid:ab,kw,ti OR 'chronic polyarthriti*':ab,kw,ti OR 'inflammatory arthrit*':ab,kw,ti OR beauvais:ab,kw,ti OR (((rheumatic OR reumatic OR revmatic OR deform*) NEAR/3 (polyarthr* OR arthr*)):ab,kw,ti) OR (ra:ab,kw,ti NOT ((((rosmarinic OR 'relative abundance' OR retinoic) NEAR/3 ra):ab,kw,ti) OR 226ra:ab,kw,ti OR '226 ra':ab,kw,ti OR radium:ab,kw,ti)))

#1

(mdhdc OR '71970 43 9' OR 'chebi:75949' OR '4-methyl-1,4-dihydro-3,5-pyridinedicarboxaldehyde' OR '3,5-diformyl-4-methyl-1,4-dihydropyridine' OR '4-methyl-1,4-dihydro-3,5-pyridinedicarbaldehyde' OR '4-methyl-1,4-dihydropyridine-3,5-dicarboxaldehyde' OR 'mda aa' OR 'malondialdehyde acetaldehyde' OR schembl6560442 OR dtxsid10500581 OR q27145654 OR '1,4-dihydro-4-methyl-3,5-pyridinedicarboxaldehyde (aci)' OR '4-methyl-1,4-dihydropyridine-3,5-dialdehyde' OR '4-methyl-1,4-dihydropyridine-3,5-dicarbaldehyde' OR (epitope NEAR/3 190359) OR (((maa NEAR/5 (adduct* OR anti OR antigen* OR antibod* OR epitope* OR immunoglob* OR igg OR igm OR iga OR ige OR ig OR 'immune globulin*' OR modif* OR comodif* OR 'post translational' OR alter* OR lysine OR terminal OR adduct* OR albumin OR collagen OR vimentin OR mgp OR 'matrix gla protein' OR fibrinogen OR 'cross link*' OR autoantibod* OR 'self protein*' OR oxidat* OR ldl OR hdl OR vldl OR lipoprotein*)):ab,kw,ti) NOT ((maa OR maas) NEAR/2 ('myositis-associated' OR 'macro-aggregated albumin' OR 'macroaggregated albumin')):ab,kw,ti)) AND ((aia:ab,kw,ti OR caia:ab,kw,ti OR cia:ab,kw,ti OR pia:ab,kw,ti OR (((experimental OR acpa OR aia OR mycobacteri* OR 'antigen induced' OR 'anti citrullin*' OR 'citrullinated-protein' OR 'anti gpi' OR antigpi OR 'glycoprotein i' OR 'anti gp1' OR antigp1 OR 'glycoprotein 1' OR 'gp i' OR 'gp 1' OR gpi OR gp1 OR cii OR collagen OR zymosan OR 'methylated bsa' OR 'methylated bovine serum albumin' OR mbsa OR 'streptococcal cell wall' OR 'bacterial cell wall' OR scw OR 'serum transfer' OR 'transferred serum' OR 'glucose-6-phosphate isomerase' OR antig6pi OR g6pi OR htnf OR 'tumor necrosis factor' OR 'tnf tg' OR 'htnf tg' OR tnf* OR tg3647 OR 'k/bxn' OR krnxnod OR skg OR ts1xhacii OR hemagglutinin OR d1cc OR 'major histocompatibility complex class ii' OR 'mhc ii' OR 'mhc class ii' OR 'class ii mhc' OR tcr OR 't-cell receptor*' OR 'organic dust*' OR 'agricultural dust*' OR ode OR pristane) NEAR/3 (arthriti* OR joint* OR synov* OR cartilag* OR knee* OR 'foot pad*' OR foot OR feet OR ankle* OR paw*)):ab,kw,ti)) AND ('animal'/exp OR balb*:ab,kw,ti,de OR 'nu/nu':ab,kw,ti,de OR dba*:ab,kw,ti,de OR b10*:ab,kw,ti,de OR c57bl*:ab,kw,ti,de OR nod:ab,kw,ti,de OR krnxnod:ab,kw,ti,de OR fvb:ab,kw,ti,de OR 'k/bxn':ab,kw,ti,de OR skg:ab,kw,ti,de OR ts1xhacii:ab,kw,ti,de OR 'd1cc transgenic':ab,kw,ti,de OR mice:ab,kw,ti,de OR mouse:ab,kw,ti,de OR murin*:ab,kw,ti,de OR murid*:ab,kw,ti,de OR mus:ab,kw,ti,de OR rat:ab,kw,ti,de OR rats:ab,kw,ti,de OR rodent*:ab,kw,ti,de OR rattus:ab,kw,ti,de OR wistar:ab,kw,ti,de OR lewis:ab,kw,ti,de OR agouti:ab,kw,ti,de OR ewe*:ab,kw,ti,de OR merino:ab,kw,ti,de OR lamb*:ab,kw,ti,de OR sheep:ab,kw,ti,de OR ovine:ab,kw,ti,de OR ovis:ab,kw,ti,de OR 'non human primate*':ab,kw,ti,de OR anthropoid*:ab,kw,ti,de OR monkey*:ab,kw,ti,de OR rhesus:ab,kw,ti,de OR paw*:ab,kw,ti,de) OR (('spontaneous arthriti*' NEAR/3 ('non-human primate' OR nhp OR rhesus OR monkey*)):ab,kw,ti) OR 'experimental arthritis'/exp OR 'experimental arthriti*')

 **************************************************************************************************************

**Scopus:**

Case report search:

TITLE (case W/1 (report* OR series)) AND (((((TITLE-ABS (AIA OR CAIA OR CIA OR PIA) OR AUTHKEY (AIA OR CAIA OR CIA OR PIA) OR TITLE-ABS ((experimental OR ACPA OR AIA OR mycobacteri* OR antigen-induced OR anti-citrullin* OR "citrullinated-protein" OR anti-gpi OR antigpi OR "glycoprotein I" OR anti-gp1 OR antigp1 OR "glycoprotein 1" OR "GP I" OR "GP 1" OR GPI OR GP1 OR CII OR Collagen OR Zymosan OR methylated-bsa OR "methylated bovine serum albumin" OR mbsa OR "streptococcal cell wall" OR "bacterial cell wall" OR SCW OR "serum transfer" OR "transferred serum" OR "glucose-6-phosphate isomerase" OR antig6pi OR G6PI OR htnf OR "tumor necrosis factor" OR tnf-tg OR htnf-tg OR tnf* OR Tg3647 OR "k/bxn" OR KRNxNOD OR SKG OR TS1xHACII OR hemagglutinin OR D1CC OR "major histocompatibility complex Class II" OR MHC-II OR MHC-class-II OR "class II MHC" OR TCR OR "t-cell receptor*" OR "organic dust*" OR "agricultural dust*" OR ODE OR pristane) W/3 (arthriti* OR joint* OR synov* OR cartilag* OR knee* OR "foot pad*" OR foot OR feet OR ankle* OR paw*)) OR AUTHKEY ((experimental OR ACPA OR AIA OR mycobacteri* OR antigen-induced OR anti-citrullin* OR "citrullinated-protein" OR anti-gpi OR antigpi OR "glycoprotein I" OR anti-gp1 OR antigp1 OR "glycoprotein 1" OR "GP I" OR "GP 1" OR GPI OR GP1 OR CII OR Collagen OR Zymosan OR methylated-bsa OR "methylated bovine serum albumin" OR mbsa OR "streptococcal cell wall" OR "bacterial cell wall" OR SCW OR "serum transfer" OR "transferred serum" OR "glucose-6-phosphate isomerase" OR antig6pi OR G6PI OR htnf OR "tumor necrosis factor" OR tnf-tg OR htnf-tg OR tnf* OR Tg3647 OR "k/bxn" OR KRNxNOD OR SKG OR TS1xHACII OR hemagglutinin OR D1CC OR "major histocompatibility complex Class II" OR MHC-II OR MHC-class-II OR "class II MHC" OR TCR OR "t-cell receptor*" OR "organic dust*" OR "agricultural dust*" OR ODE OR pristane) W/3 (arthriti* OR joint* OR synov* OR cartilag* OR knee* OR "foot pad*" OR foot OR feet OR ankle* OR paw*))) AND (TITLE-ABS-KEY (animal*OR balb* OR "nu/nu" OR DBA* OR B10* OR C57Bl* OR NOD OR KRNxNOD OR FVB OR "k/bxn" OR SKG OR TS1xHACII OR D1CC-transgenic OR mice OR mouse OR murin* OR murid* OR mus OR rattus OR rat OR rats OR rodent* OR rattus OR wistar OR Lewis OR agouti OR ewe* OR merino OR lamb* OR sheep OR ovine OR ovis OR non-human-primate* OR anthropoid* OR monkey* OR rhesus OR paw*)) ) OR TITLE-ABS-KEY ("Spontaneous arthriti*" W/3 ("non-human primate" OR nhp OR rhesus OR monkey*))OR TITLE-ABS-KEY ("experimental arthritis"))) AND (((mdhdc OR "71970 43 9" OR "chebi:75949" OR "4-methyl-1,4-dihydro-3,5-pyridinedicarboxaldehyde" OR "3,5-diformyl-4-methyl-1,4-dihydropyridine" OR "4-methyl-1,4-dihydro-3,5-pyridinedicarbaldehyde" OR "4-methyl-1,4-dihydropyridine-3,5-dicarboxaldehyde" OR "mda aa" OR {malondialdehyde acetaldehyde} OR schembl6560442 OR dtxsid10500581 OR q27145654 OR "1,4-dihydro-4-methyl-3,5-pyridinedicarboxaldehyde (aci)" OR "4-methyl-1,4-dihydropyridine-3,5-dialdehyde" OR "4-methyl-1,4-dihydropyridine-3,5-dicarbaldehyde" OR (epitope W/3 190359) OR ((maa W/5 (adduct* OR anti OR antigen* OR antibod* OR epitope* OR immunoglob* OR igg OR igm OR iga OR ige OR ig OR "immune globulin*" OR modif* OR comodif* OR "post translational" OR alter* OR lysine OR terminal OR adduct* OR albumin OR collagen OR vimentin OR mgp OR "matrix gla protein" OR fibrinogen OR "cross link*" OR autoantibod* OR "self protein*" OR oxidat* OR ldl OR hdl OR vldl OR lipoprotein*)) AND NOT ((maa OR maas) W/2 ("myositis-associated" OR "macro-aggregated albumin" OR "macroaggregated albumin")))) ))) OR ((TITLE-ABS (felty* OR "stills disease" OR "still's disease" OR "still disease" OR beauvais* OR caplan* OR rheumarthrit* OR reumarthrit* OR revmarthrit* OR rheumatism* OR reumatism* OR revmatism* OR rheumatoid* OR reumatoid OR revmatoid OR "chronic polyarthriti*" OR "inflammatory arthrit*" OR beauvais OR ((rheumatic OR reumatic OR revmatic OR deform*) W/3 (polyarthr* OR arthr*)) OR (ra AND NOT (((rosmarinic OR "relative abundance" OR retinoic) W/3 ra) OR 226ra OR "226 ra" OR radium)) ) OR AUTHKEY (felty* OR "stills disease" OR "still's disease" OR "still disease" OR beauvais* OR caplan* OR rheumarthrit* OR reumarthrit* OR revmarthrit* OR rheumatism* OR reumatism* OR revmatism* OR rheumatoid* OR reumatoid OR revmatoid OR "chronic polyarthriti*" OR "inflammatory arthrit*" OR beauvais OR ((rheumatic OR reumatic OR revmatic OR deform*) W/3 (polyarthr* OR arthr*)) OR (ra AND NOT (((rosmarinic OR "relative abundance" OR retinoic) W/3 ra) OR 226ra OR "226 ra" OR radium)) )) AND ((mdhdc OR "71970 43 9" OR "chebi:75949" OR "4-methyl-1,4-dihydro-3,5-pyridinedicarboxaldehyde" OR "3,5-diformyl-4-methyl-1,4-dihydropyridine" OR "4-methyl-1,4-dihydro-3,5-pyridinedicarbaldehyde" OR "4-methyl-1,4-dihydropyridine-3,5-dicarboxaldehyde" OR "mda aa" OR {malondialdehyde acetaldehyde} OR schembl6560442 OR dtxsid10500581 OR q27145654 OR "1,4-dihydro-4-methyl-3,5-pyridinedicarboxaldehyde (aci)" OR "4-methyl-1,4-dihydropyridine-3,5-dialdehyde" OR "4-methyl-1,4-dihydropyridine-3,5-dicarbaldehyde" OR (epitope W/3 190359) OR ((maa W/5 (adduct* OR anti OR antigen* OR antibod* OR epitope* OR immunoglob* OR igg OR igm OR iga OR ige OR ig OR "immune globulin*" OR modif* OR comodif* OR "post translational" OR alter* OR lysine OR terminal OR adduct* OR albumin OR collagen OR vimentin OR mgp OR "matrix gla protein" OR fibrinogen OR "cross link*" OR autoantibod* OR "self protein*" OR oxidat* OR ldl OR hdl OR vldl OR lipoprotein*)) AND NOT ((maa OR maas) W/2 ("myositis-associated" OR "macro-aggregated albumin" OR "macroaggregated albumin"))))))AND TITLE (case W/1 (report* OR series)) AND ( EXCLUDE ( SRCTYPE,"k" ) ) AND ( EXCLUDE ( DOCTYPE,"re" ) OR EXCLUDE ( DOCTYPE,"ed" ) OR EXCLUDE ( DOCTYPE,"ch" ) ) AND ( LIMIT-TO ( LANGUAGE,"English" ) )

Other search:

(((((TITLE-ABS (AIA OR CAIA OR CIA OR PIA) OR AUTHKEY (AIA OR CAIA OR CIA OR PIA) OR TITLE-ABS ((experimental OR ACPA OR AIA OR mycobacteri* OR antigen-induced OR anti-citrullin* OR "citrullinated-protein" OR anti-gpi OR antigpi OR "glycoprotein I" OR anti-gp1 OR antigp1 OR "glycoprotein 1" OR "GP I" OR "GP 1" OR GPI OR GP1 OR CII OR Collagen OR Zymosan OR methylated-bsa OR "methylated bovine serum albumin" OR mbsa OR "streptococcal cell wall" OR "bacterial cell wall" OR SCW OR "serum transfer" OR "transferred serum" OR "glucose-6-phosphate isomerase" OR antig6pi OR G6PI OR htnf OR "tumor necrosis factor" OR tnf-tg OR htnf-tg OR tnf* OR Tg3647 OR "k/bxn" OR KRNxNOD OR SKG OR TS1xHACII OR hemagglutinin OR D1CC OR "major histocompatibility complex Class II" OR MHC-II OR MHC-class-II OR "class II MHC" OR TCR OR "t-cell receptor*" OR "organic dust*" OR "agricultural dust*" OR ODE OR pristane) W/3 (arthriti* OR joint* OR synov* OR cartilag* OR knee* OR "foot pad*" OR foot OR feet OR ankle* OR paw*)) OR AUTHKEY ((experimental OR ACPA OR AIA OR mycobacteri* OR antigen-induced OR anti-citrullin* OR "citrullinated-protein" OR anti-gpi OR antigpi OR "glycoprotein I" OR anti-gp1 OR antigp1 OR "glycoprotein 1" OR "GP I" OR "GP 1" OR GPI OR GP1 OR CII OR Collagen OR Zymosan OR methylated-bsa OR "methylated bovine serum albumin" OR mbsa OR "streptococcal cell wall" OR "bacterial cell wall" OR SCW OR "serum transfer" OR "transferred serum" OR "glucose-6-phosphate isomerase" OR antig6pi OR G6PI OR htnf OR "tumor necrosis factor" OR tnf-tg OR htnf-tg OR tnf* OR Tg3647 OR "k/bxn" OR KRNxNOD OR SKG OR TS1xHACII OR hemagglutinin OR D1CC OR "major histocompatibility complex Class II" OR MHC-II OR MHC-class-II OR "class II MHC" OR TCR OR "t-cell receptor*" OR "organic dust*" OR "agricultural dust*" OR ODE OR pristane) W/3 (arthriti* OR joint* OR synov* OR cartilag* OR knee* OR "foot pad*" OR foot OR feet OR ankle* OR paw*))) AND (TITLE-ABS-KEY (animal*OR balb* OR "nu/nu" OR DBA* OR B10* OR C57Bl* OR NOD OR KRNxNOD OR FVB OR "k/bxn" OR SKG OR TS1xHACII OR D1CC-transgenic OR mice OR mouse OR murin* OR murid* OR mus OR rattus OR rat OR rats OR rodent* OR rattus OR wistar OR Lewis OR agouti OR ewe* OR merino OR lamb* OR sheep OR ovine OR ovis OR non-human-primate* OR anthropoid* OR monkey* OR rhesus OR paw*)) ) OR TITLE-ABS-KEY ("Spontaneous arthriti*" W/3 ("non-human primate" OR nhp OR rhesus OR monkey*))OR TITLE-ABS-KEY ("experimental arthritis"))) AND (((mdhdc OR "71970 43 9" OR "chebi:75949" OR "4-methyl-1,4-dihydro-3,5-pyridinedicarboxaldehyde" OR "3,5-diformyl-4-methyl-1,4-dihydropyridine" OR "4-methyl-1,4-dihydro-3,5-pyridinedicarbaldehyde" OR "4-methyl-1,4-dihydropyridine-3,5-dicarboxaldehyde" OR "mda aa" OR {malondialdehyde acetaldehyde} OR schembl6560442 OR dtxsid10500581 OR q27145654 OR "1,4-dihydro-4-methyl-3,5-pyridinedicarboxaldehyde (aci)" OR "4-methyl-1,4-dihydropyridine-3,5-dialdehyde" OR "4-methyl-1,4-dihydropyridine-3,5-dicarbaldehyde" OR (epitope W/3 190359) OR ((maa W/5 (adduct* OR anti OR antigen* OR antibod* OR epitope* OR immunoglob* OR igg OR igm OR iga OR ige OR ig OR "immune globulin*" OR modif* OR comodif* OR "post translational" OR alter* OR lysine OR terminal OR adduct* OR albumin OR collagen OR vimentin OR mgp OR "matrix gla protein" OR fibrinogen OR "cross link*" OR autoantibod* OR "self protein*" OR oxidat* OR ldl OR hdl OR vldl OR lipoprotein*)) AND NOT ((maa OR maas) W/2 ("myositis-associated" OR "macro-aggregated albumin" OR "macroaggregated albumin")))) ))) OR ((TITLE-ABS (felty* OR "stills disease" OR "still's disease" OR "still disease" OR beauvais* OR caplan* OR rheumarthrit* OR reumarthrit* OR revmarthrit* OR rheumatism* OR reumatism* OR revmatism* OR rheumatoid* OR reumatoid OR revmatoid OR "chronic polyarthriti*" OR "inflammatory arthrit*" OR beauvais OR ((rheumatic OR reumatic OR revmatic OR deform*) W/3 (polyarthr* OR arthr*)) OR (ra AND NOT (((rosmarinic OR "relative abundance" OR retinoic) W/3 ra) OR 226ra OR "226 ra" OR radium)) ) OR AUTHKEY (felty* OR "stills disease" OR "still's disease" OR "still disease" OR beauvais* OR caplan* OR rheumarthrit* OR reumarthrit* OR revmarthrit* OR rheumatism* OR reumatism* OR revmatism* OR rheumatoid* OR reumatoid OR revmatoid OR "chronic polyarthriti*" OR "inflammatory arthrit*" OR beauvais OR ((rheumatic OR reumatic OR revmatic OR deform*) W/3 (polyarthr* OR arthr*)) OR (ra AND NOT (((rosmarinic OR "relative abundance" OR retinoic) W/3 ra) OR 226ra OR "226 ra" OR radium)) )) AND ((mdhdc OR "71970 43 9" OR "chebi:75949" OR "4-methyl-1,4-dihydro-3,5-pyridinedicarboxaldehyde" OR "3,5-diformyl-4-methyl-1,4-dihydropyridine" OR "4-methyl-1,4-dihydro-3,5-pyridinedicarbaldehyde" OR "4-methyl-1,4-dihydropyridine-3,5-dicarboxaldehyde" OR "mda aa" OR {malondialdehyde acetaldehyde} OR schembl6560442 OR dtxsid10500581 OR q27145654 OR "1,4-dihydro-4-methyl-3,5-pyridinedicarboxaldehyde (aci)" OR "4-methyl-1,4-dihydropyridine-3,5-dialdehyde" OR "4-methyl-1,4-dihydropyridine-3,5-dicarbaldehyde" OR (epitope W/3 190359) OR ((maa W/5 (adduct* OR anti OR antigen* OR antibod* OR epitope* OR immunoglob* OR igg OR igm OR iga OR ige OR ig OR "immune globulin*" OR modif* OR comodif* OR "post translational" OR alter* OR lysine OR terminal OR adduct* OR albumin OR collagen OR vimentin OR mgp OR "matrix gla protein" OR fibrinogen OR "cross link*" OR autoantibod* OR "self protein*" OR oxidat* OR ldl OR hdl OR vldl OR lipoprotein*)) AND NOT ((maa OR maas) W/2 ("myositis-associated" OR "macro-aggregated albumin" OR "macroaggregated albumin")))))) AND NOT TITLE (case W/1 (report* OR series)) AND ( EXCLUDE ( SRCTYPE,"k" ) ) AND ( EXCLUDE ( DOCTYPE,"re" ) OR EXCLUDE ( DOCTYPE,"ed" ) OR EXCLUDE ( DOCTYPE,"ch" ) ) AND ( LIMIT-TO ( LANGUAGE,"English" ) )

 **************************************************************************************************************

**SciFinder:**

#1 Abstract/keyword

Animal OR animals OR balb* OR “nu/nu” OR dba OR b10 OR c57bl6 OR c57bl OR nod OR krnxnod OR fvb OR “k/bxn” OR skg OR ts1xhacii OR “d1cc transgenic” OR mice OR mouse OR murin* OR muridae OR mus OR rat OR rats OR rodentia OR rodents OR rodent OR rattus OR wistar OR lewis OR agouti OR ewe OR ewes OR merino OR lamb OR lambs OR sheep OR ovine OR ovis OR “non human primate” OR “non human primates” OR anthropoidea OR monkey OR monkeys OR rhesus OR paw OR paws

AND

Arthritis OR arthritides OR joint OR joints OR synovitis OR synovitides OR synovial OR cartilage OR knee OR knees OR foot OR feet OR ankle OR ankles OR paw OR paws

AND

experimental OR acpa OR aia OR mycobacteria OR “antigen induced” OR “anti citrullinated” OR “citrullinated-protein” OR “anti gpi” OR antigpi OR “glycoprotein i” OR “anti gp1” OR antigp1 OR “glycoprotein 1” OR “gp i” OR “gp 1” OR gpi OR gp1 OR cii OR collagen OR zymosan OR “methylated bsa” OR “methylated bovine serum albumin” OR mbsa OR “streptococcal cell wall” OR “bacterial cell wall” OR scw OR “serum transfer” OR “transferred serum” OR “glucose-6-phosphate isomerase” OR antig6pi OR g6pi OR htnf OR “tumor necrosis factor” OR “tnf tg” OR “htnf tg” OR tnf OR tg3647 OR “kbxn” OR bxn OR krnxnod OR skg OR ts1xhacii OR hemagglutinin OR d1cc OR “major histocompatibility complex class ii” OR “mhc ii” OR “mhc class ii” OR “class ii mhc” OR tcr OR “t-cell receptor” OR “t-cell receptors” OR “organic dust” OR “agricultural dust” OR ode OR pristane

AND

mdhdc OR 71970-43-9 OR “mda aa” OR “malondialdehyde acetaldehyde” OR schembl6560442 OR dtxsid10500581 OR q27145654

#2 Abstract/keyword

“spontaneous arthritis” OR “spontaneous arthritides”

AND

“non-human primate” OR “non-human primates” OR nhp OR rhesus OR monkey OR monkeys

AND

mdhdc OR 71970-43-9 OR “mda aa” OR “malondialdehyde acetaldehyde” OR schembl6560442 OR dtxsid10500581 OR q27145654

#3 Abstract/keyword

aia OR caia OR cia OR pia OR “experimental arthritis” OR “experimental arthritides”

AND

mdhdc OR 71970-43-9 OR “mda aa” OR “malondialdehyde acetaldehyde” OR schembl6560442 OR dtxsid10500581 OR q27145654

#4 Abstract/keyword

mdhdc OR 71970-43-9 OR “mda aa” OR “malondialdehyde acetaldehyde” OR schembl6560442 OR dtxsid10500581 OR q27145654

AND

felty OR felty’s OR “stills disease” OR “still’s disease” OR “still disease” OR beauvais OR caplan OR caplan’s OR rheumarthrit* OR reumarthrit* OR revmarthrit* OR rheumatism OR reumatism OR revmatism OR rheumatoid OR reumatoid OR revmatoid OR “chronic polyarthritis” OR “chronic polyarthritides” OR “inflammatory arthritides” OR “inflammatory arthritis”

#5

removed editorials/reviews from #1 OR #2 OR #3 OR #4

 **************************************************************************************************************

**Cochrane Library   (via Wiley)**

ID         Search Hits

#1        MeSH descriptor: [Arthritis, Experimental] explode all trees

#2        MeSH descriptor: [Arthritis] this term only

#3        MeSH descriptor: [Arthritis, Rheumatoid] explode all trees

#4        MeSH descriptor: [Animals] explode all trees

#5        #4 OR  balb*:ab,kw,ti OR "nu/nu":ab,kw,ti OR dba*:ab,kw,ti OR b10*:ab,kw,ti OR c57bl*:ab,kw,ti OR nod:ab,kw,ti OR krnxnod:ab,kw,ti OR fvb:ab,kw,ti OR "k/bxn":ab,kw,ti OR skg:ab,kw,ti OR ts1xhacii:ab,kw,ti OR "d1cc transgenic":ab,kw,ti OR mice:ab,kw,ti OR mouse:ab,kw,ti OR murin*:ab,kw,ti OR murid*:ab,kw,ti OR mus:ab,kw,ti OR rat:ab,kw,ti OR rats:ab,kw,ti OR rodent*:ab,kw,ti OR rattus:ab,kw,ti OR wistar:ab,kw,ti OR lewis:ab,kw,ti OR agouti:ab,kw,ti OR ewe*:ab,kw,ti OR merino:ab,kw,ti OR lamb*:ab,kw,ti OR sheep:ab,kw,ti OR ovine:ab,kw,ti OR ovis:ab,kw,ti OR "non human primate":ab,kw,ti OR "non human primates":ab,kw,ti OR anthropoid:ab,kw,ti OR anthropoidea:ab,kw,ti OR monkey*:ab,kw,ti OR rhesus:ab,kw,ti OR paw*:ab,kw,ti

#6        (("spontaneous arthritis" OR "spontaneous arthritides" ) NEAR/3 ("non-human primate" OR nhp OR rhesus OR monkey*)):ab,kw,ti OR "experimental arthritis" OR "experimental arthritides" OR #1

#7        (mdhdc OR "71970 43 9" OR "chebi:75949" OR "4-methyl-1,4-dihydro-3,5-pyridinedicarboxaldehyde" OR "3,5-diformyl-4-methyl-1,4-dihydropyridine" OR "4-methyl-1,4-dihydro-3,5-pyridinedicarbaldehyde" OR "4-methyl-1,4-dihydropyridine-3,5-dicarboxaldehyde" OR "mda aa" OR "malondialdehyde acetaldehyde" OR schembl6560442 OR dtxsid10500581 OR q27145654 OR "1,4-dihydro-4-methyl-3,5-pyridinedicarboxaldehyde (aci)" OR "4-methyl-1,4-dihydropyridine-3,5-dialdehyde" OR "4-methyl-1,4-dihydropyridine-3,5-dicarbaldehyde" OR (epitope NEAR/3 190359) OR (((maa NEAR/5 (adduct* OR anti OR antigen* OR antibod* OR epitope* OR immunoglob* OR igg OR igm OR iga OR ige OR ig OR "immune globulin" OR "immune globulins" OR modif* OR comodif* OR "post translational" OR alter* OR lysine OR terminal OR adduct* OR albumin OR collagen OR vimentin OR mgp OR "matrix gla protein" OR fibrinogen OR "cross link" OR "cross linked" OR "cross linking" OR "cross links" OR autoantibod* OR "self protein" OR "self proteins" OR oxidat* OR ldl OR hdl OR vldl OR lipoprotein*)):ab,kw,ti) NOT ((maa OR maas) NEAR/2 ("myositis-associated" OR "macro-aggregated albumin" OR "macroaggregated albumin")):ab,kw,ti))

#8        (aia:ab,kw,ti OR caia:ab,kw,ti OR cia:ab,kw,ti OR pia:ab,kw,ti OR (((experimental OR acpa OR aia OR mycobacteri* OR "antigen induced" OR "anti citrullinated" OR "citrullinated-protein" OR "anti gpi" OR antigpi OR "glycoprotein i" OR "anti gp1" OR antigp1 OR "glycoprotein 1" OR "gp i" OR "gp 1" OR gpi OR gp1 OR cii OR collagen OR zymosan OR "methylated bsa" OR "methylated bovine serum albumin" OR mbsa OR "streptococcal cell wall" OR "bacterial cell wall" OR scw OR "serum transfer" OR "transferred serum" OR "glucose-6-phosphate isomerase" OR antig6pi OR g6pi OR htnf OR "tumor necrosis factor" OR "tnf tg" OR "htnf tg" OR tnf* OR tg3647 OR "k/bxn" OR krnxnod OR skg OR ts1xhacii OR hemagglutinin OR d1cc OR "major histocompatibility complex class ii" OR "mhc ii" OR "mhc class ii" OR "class ii mhc" OR tcr OR "t-cell receptor" OR "t-cell receptors" OR "organic dust" OR "organic dusts" OR "agricultural dust" OR "agricultural dusts" OR ode OR pristane) NEAR/3 (arthriti* OR joint* OR synov* OR cartilag* OR knee* OR "foot pad" OR "foot pads" OR foot OR feet OR ankle* OR paw*)):ab,kw,ti))

#9        ((#5 AND #8) OR #6) AND #7

#10      (#3 OR #4 OR felty*:ab,kw,ti OR "stills disease":ab,kw,ti OR "still's disease":ab,kw,ti OR "still disease":ab,kw,ti OR beauvais*:ab,kw,ti OR caplan*:ab,kw,ti OR rheumarthrit*:ab,kw,ti OR reumarthrit*:ab,kw,ti OR revmarthrit*:ab,kw,ti OR rheumatism*:ab,kw,ti OR reumatism*:ab,kw,ti OR revmatism*:ab,kw,ti OR rheumatoid*:ab,kw,ti OR reumatoid:ab,kw,ti OR revmatoid:ab,kw,ti OR "chronic polyarthritis":ab,kw,ti OR "inflammatory arthritis":ab,kw,ti OR "chronic polyarthritides":ab,kw,ti OR "inflammatory arthritides":ab,kw,ti OR beauvais:ab,kw,ti OR (((rheumatic OR reumatic OR revmatic OR deform*) NEAR/3 (polyarthr* OR arthr*)):ab,kw,ti) OR (ra:ab,kw,ti NOT ((((rosmarinic OR "relative abundance" OR retinoic) NEAR/3 ra):ab,kw,ti) OR 226ra:ab,kw,ti OR "226 ra":ab,kw,ti OR radium:ab,kw,ti)))

#11      #7 AND #10

#12      #9 OR #11
